# Evaluation of Candidate Stromal Epithelial Cross-Talk Genes Identifies Association between Risk of Serous Ovarian Cancer and *TERT*, a Cancer Susceptibility “Hot-Spot”

**DOI:** 10.1371/journal.pgen.1001016

**Published:** 2010-07-08

**Authors:** Sharon E. Johnatty, Jonathan Beesley, Xiaoqing Chen, Stuart Macgregor, David L. Duffy, Amanda B. Spurdle, Anna deFazio, Natalie Gava, Penelope M. Webb, Australian Ovarian Cancer Study Group, Australian Cancer Study (Ovarian Cancer), Mary Anne Rossing, Jennifer Anne Doherty, Marc T. Goodman, Galina Lurie, Pamela J. Thompson, Lynne R. Wilkens, Roberta B. Ness, Kirsten B. Moysich, Jenny Chang-Claude, Shan Wang-Gohrke, Daniel W. Cramer, Kathryn L. Terry, Susan E. Hankinson, Shelley S. Tworoger, Montserrat Garcia-Closas, Hannah Yang, Jolanta Lissowska, Stephen J. Chanock, Paul D. Pharoah, Honglin Song, Alice S. Whitemore, Celeste L. Pearce, Daniel O. Stram, Anna H. Wu, Malcolm C. Pike, Simon A. Gayther, Susan J. Ramus, Usha Menon, Aleksandra Gentry-Maharaj, Hoda Anton-Culver, Argyrios Ziogas, Estrid Hogdall, Susanne K. Kjaer, Claus Hogdall, Andrew Berchuck, Joellen M. Schildkraut, Edwin S. Iversen, Patricia G. Moorman, Catherine M. Phelan, Thomas A. Sellers, Julie M. Cunningham, Robert A. Vierkant, David N. Rider, Ellen L. Goode, Izhak Haviv, Georgia Chenevix-Trench, Ovarian Cancer Association Consortium

**Affiliations:** 1 Queensland Institute of Medical Research, Brisbane, Australia; 2 Department of Gynaecological Oncology and Westmead Institute for Cancer Research, University of Sydney at the Westmead Millennium Institute, Westmead Hospital, Sydney, Australia; 3 Peter MacCallum Cancer Centre, East Melbourne, Australia; 4 Epidemiology Program, Division of Public Health Services, Fred Hutchinson Cancer Research Center, Seattle, Washington, United States of America; 5 Cancer Research Center of Hawaii, University of Hawaii, Hilo, Hawaii, United States of America; 6 University of Texas School of Public Health, Houston, Texas, United States of America; 7 Roswell Park Cancer Center, Buffalo, New York, United States of America; 8 Unit of Genetic Epidemiology, Division of Cancer Epidemiology, Deutsches Krebsforschungszentrum, Heidelberg, Germany; 9 Department of Obstetrics and Gynecology, University of Ulm, Ulm, Germany; 10 Obstetrics and Gynecology Epidemiology Center, Brigham and Women's Hospital, Boston, Massachusetts, United States of America; 11 Channing Laboratory, Department of Medicine, Brigham and Women's Hospital and Harvard Medical School, Boston, Massachusetts, United States of America; 12 Department of Epidemiology, Harvard School of Public Health, Boston, Massachusetts, United States of America; 13 National Cancer Institute, Division of Cancer Epidemiology and Genetics, Rockville, Maryland, United States of America; 14 Department of Cancer Epidemiology and Prevention, The M. Sklodowska-Curie Cancer Center and Institute of Oncology, Warsaw, Poland; 15 Laboratory of Translational Genomics Division of Cancer Epidemiology and Genetics National Cancer Institute and Core Genotyping Facility Division of Cancer Epidemiology and Genetics National Cancer Institute, Bethesda, Maryland, United States of America; 16 Department of Oncology and Public Health and Primary Care, University of Cambridge, Cambridge, United Kingdom; 17 Department of Health Research and Policy, Stanford University School of Medicine, Stanford, California, United States of America; 18 Department of Preventative Medicine, Keck School of Medicine, University of Southern California Norris Comprehensive Cancer Center, Los Angeles, California, United States of America; 19 Department of Gynaecological Oncology, UCL EGA Institute for Women's Health, University College London, London, United Kingdom; 20 Department of Epidemiology, School of Medicine, University of California Irvine, Irvine, California, United States of America; 21 Department of Virus, Hormones, and Cancer, Institute of Cancer Epidemiology, Danish Cancer Society, Copenhagen, Denmark; 22 Gynaecologic Clinic, The Juliane Marie Centre, Righosptalet, University of Copenhagen, Copenhagen, Denmark; 23 Division of Preventative Medicine, The Duke Comprehensive Cancer Center, Durham, North Carolina, United States of America; 24 Division of Cancer Prevention and Control, H. Lee Moffitt Cancer Center and Research Institute, Magnolia Drive, Tampa, Florida, United States of America; 25 Department of Health Sciences Research, Mayo Clinic College of Medicine, Rochester, Minnesota, United States of America; 26 The Blood and DNA Profiling Facility, Baker IDI, Melbourne, Australia; 27 Department of Biochemistry, School of Medicine, University of Melbourne, Parkville, Australia; University of Geneva Medical School, Switzerland

## Abstract

We hypothesized that variants in genes expressed as a consequence of interactions between ovarian cancer cells and the host micro-environment could contribute to cancer susceptibility. We therefore used a two-stage approach to evaluate common single nucleotide polymorphisms (SNPs) in 173 genes involved in stromal epithelial interactions in the Ovarian Cancer Association Consortium (OCAC). In the discovery stage, cases with epithelial ovarian cancer (n = 675) and controls (n = 1,162) were genotyped at 1,536 SNPs using an Illumina GoldenGate assay. Based on Positive Predictive Value estimates, three SNPs—*PODXL* rs1013368, *ITGA6* rs13027811, and *MMP3* rs522616—were selected for replication using TaqMan genotyping in up to 3,059 serous invasive cases and 8,905 controls from 16 OCAC case-control studies. An additional 18 SNPs with *P*_per-allele_<0.05 in the discovery stage were selected for replication in a subset of five OCAC studies (n = 1,233 serous invasive cases; n = 3,364 controls). The discovery stage associations in *PODXL*, *ITGA6*, and *MMP3* were attenuated in the larger replication set (adj. *P*_per-allele_≥0.5). However genotypes at *TERT* rs7726159 were associated with ovarian cancer risk in the smaller, five-study replication study (*P*_per-allele_ = 0.03). Combined analysis of the discovery and replication sets for this *TERT* SNP showed an increased risk of serous ovarian cancer among non-Hispanic whites [adj. OR_per-allele_ 1.14 (1.04–1.24) *p* = 0.003]. Our study adds to the growing evidence that, like the 8q24 locus, the telomerase reverse transcriptase locus at 5p15.33, is a general cancer susceptibility locus.

## Author Summary

In this article, we report the findings from a large-scale analysis of common variation in genes that are expressed as a consequence of interactions between ovarian cancer cells and their host micro-environment that could influence serous ovarian cancer risk. We evaluated 1,302 common variants within or near 173 genes in two large case-control studies from the Ovarian Cancer Association Consortium (OCAC) and selected three variants for further evaluation in sixteen OCAC studies and an additional 18 for evaluation in five OCAC studies. We observed a significantly increased risk of serous ovarian cancer associated with a variant in the telomerase reverse transcriptase (*TERT*) gene. Although *TERT* variants have not been previously shown to contribute to ovarian cancer risk, several studies have recently reported associations between *TERT* variants and other forms of cancer, including gliomas, lung cancer, adenocarcinoma, basal cell carcinoma, prostate cancer, and multiple other cancers. *TERT* encodes a protein that is essential for the replication and maintenance of chromosomal integrity during cell division. In cancer cells, *TERT* has been linked to genomic instability and tumour cell proliferation. Further studies are necessary to confirm our findings and to investigate the mechanisms for the observed association.

## Introduction

Ovarian cancer is the seventh leading cause of cancer mortality among women globally, accounting for 4.2% of cancer deaths [1], due in part to the lack of practical screening methods and detectable symptoms in the early stages of tumor progression [2]. Although the aetiology of ovarian cancer has not been fully elucidated, it is generally agreed that family history of ovarian or breast cancer is the most important risk factor for epithelial ovarian cancer [3]. Hereditary ovarian cancer occurring in breast/ovarian cancer families has been linked to mutations in the *BRCA1* and *BRCA2* genes, while cases occurring in association with Lynch syndrome have been linked to mutations in *MSH2* and *MLH1* [4], [5]. Given that only 3% to 5% of ovarian cancer cases present from high-risk families and residual family history associations [2], it is likely that several low-penetrance genes with relatively common alleles that confer slightly increased risk may account for a portion of the risk of non-familial ovarian cancer. The Ovarian Cancer Association Consortium (OCAC) was established in 2005 to provide a forum for the identification and validation of common low-penetrance ovarian cancer susceptibility polymorphisms with increased power [6]. OCAC recently conducted a genome-wide association study (GWAS) and identified the first susceptibility locus associated with invasive ovarian cancer risk [7].

A number of hypotheses have been put forward to explain the pathogenesis of ovarian cancer [8], [9], including that of incessant ovulation which causes repeated minor trauma to the surface of the ovary, leading to proliferation of ovarian epithelium and repair of the ovulatory wound [10]. However, it has also been hypothesized that fallopian tube epithelial cells migrating to the ovulatory wound could serve as precursors to ovarian cancer [11]. Research in the past two decades compellingly suggests that the neighbors of cancer cells, collectively termed stroma, are not uninvolved bystanders [12] and studies involving three-dimensional cell culture models underscore the involvement of the extracellular matrix surrounding cancer cells in the signalling pathways that promote cell survival [13]. Fibroblasts with a carcinoma-promoting phenotype [carcinoma-associated fibroblasts (CAFs)] residing in the breast cancer microenvironment lack the ability of normal fibroblasts to attenuate the growth of neighbouring transformed epithelial cells [14]. In addition, xenograft models have shown that CAFs accelerate cancer progression through their ability to secrete stromal cell-derived factor 1 [15]. Furthermore, expression profiling of ovarian tumor samples has identified a group of high-grade invasive cancers characterized by a reactive stromal gene expression signature and extensive desmoplasia, which confer an inherently poor prognosis [16]. If this CAF-dependent model of tumorigenesis is correct, it assigns a key role to the neighboring stroma in cancer initiation.

We therefore hypothesized that subtle variation in the expression or function of genes expressed as a consequence of interactions between ovarian cancer cells and the host micro-environment could contribute to ovarian cancer susceptibility. We used a two-stage approach to comprehensively evaluate common variation in 173 genes selected for their putative role in stromal-epithelial interactions using a tagging-SNP approach and data from sixteen case-control studies participating in the Ovarian Cancer Association Consortium (OCAC).

## Results

Candidate gene selection and justification are provided in Text S1 and Table S1. Characteristics of all case-control studies that contributed data to discovery and replication analyses are provided in Table S2. Comparison of the mean age at diagnosis for cases and age at interview for controls showed that cases were significantly older compared to controls (*p*<0.05). Figure S1 provides an overview of SNP and cases-controls numbers analysed in the discovery and replication stages of this study. Discovery samples consisted of serous invasive cases from the AUS (550 cases and 1,101 controls) and MAY (125 cases and 61 controls; all non-Hispanic Whites) studies. AUS participants were not selected for ethnicity, but comprised of predominantly non-Hispanic White women. Of the 1,837 women with genotype data, three were excluded by PLINK default thresholds because >10% of SNPs failed genotyping for these individuals. Of the 1,536 single nucleotide polymorphisms (SNPs) genotyped, 1,309 SNPs passed our initial quality control (QC) criteria, and of these, seven were excluded by PLINK default thresholds. The remaining 1,302 SNPs were subject to further pruning as follows: 37 SNPs with significantly different frequencies of missing genotype data between cases and controls (*P*_Miss_<0.05); 296 SNPs with duplicate discordance and/or failure to meet Hardy-Weinberg equilibrium (HWE) criteria (0.001<*P_HWE_*<0.05). Of the remaining 969 SNPs analysed in the discovery stage, 59 SNPs with *P*_Trend_<0.05 were considered for the replication study (see Table S3).

Based on positive predictive value (PPV) estimates, the three SNPs selected for replication using TaqMan genotyping by the 16 OCAC studies were *PODXL* (podocalyxin-like) rs1013368 (PPV 33.1%), *ITGA6* (integrin, alpha 6) rs13027811 (PPV 4.5%) and *MMP3* (matrix metallopeptidase 3) rs522616 (PPV 4.4%) (Table 1). These 16 OCAC studies included all histologic subtypes, and ethnicities. An additional 18 SNPs with *P*_Trend_<0.05 which fitted into the iPLEX design were selected for replication by a subset of five of the 16 OCAC studies [AUS (additional samples not in the discovery set), MAL, SEA, UKO, and USC]. *FGF2* rs17473132 included among the 18 selected SNPs (*P*_Trend_ = 0.008) has been previously reported elsewhere [17] and is therefore excluded from this report. Replication sample sizes varied by SNP depending on which participating OCAC study met QC criteria; MAY, NCO, NEC and NHS failed QC for *PODXL* rs1013368, and GER and STA failed QC for *ITGA6* rs13027811. Table 2 provides the risk estimates adjusted for age and study site for SNPs included in the replication analysis. There was no evidence of between-study heterogeneity for any replication SNP with the exception of *TERT* rs7726159 (*p* = 0.005) (Table S4). Further examination of the site-specific Odds Ratios (ORs) showed that this was driven in part by the smaller USC study, the exclusion of which resulted in a *p*-value for between-study heterogeneity of 0.09. The associations observed in the discovery set for the three SNPs selected based on PPV values (*PODXL* rs1013368, *ITGA6* rs13027811, and *MMP3* rs522616), were completely attenuated in the larger replication analysis of 16 case control studies (adj. *P*_per-allele_≥0.5) (Table 2).

**Table 1:** Discovery analysis: risk estimates for serous ovarian cancer for three SNPs selected for replication by 16 OCAC studies.

Gene symbol	CHR	SNP	Minor Allele	Major Allele	aMAF	a *P*_HWE_	bOR	(95% CI)	b *P*_allelic_	c *P*_Trend_	dPower	ePPV
*PODXL*	7	rs1013368	G	A	0.34	1.00	1.32	(1.14–1.51)	0.0001126	0.0001037	0.51	33.1%
*ITGA6*	2	rs13027811	G	A	0.12	0.87	0.68	(0.54–0.85)	0.0008275	0.0008566	0.40	4.5%
*MMP3*	11	rs522616	G	A	0.23	0.93	0.76	(0.64–0.90)	0.001178	0.001184	0.55	4.4%

^**a**^MAF and *P*_HWE_ derived from controls.

^**b**^Odds ratios, 95% CI and p-values are derived from the allelic test for association using χ^2^ test on 1 df.

^**c**^Cochran-Armitage trend test (1df).

^**d**^Power of the study to detect the association.

^**e**^Positive predictive value.

**Table 2:** Replication analysis: risk estimates for serous invasive ovarian cancer in non-Hispanic whites for SNPs selected for replication by indicated OCAC sites.

Gene	SNP	MAF^a^	Controls	Cases	OR_Het_ b	(95% CI)	*P*	OR_Hom_ b	(95% CI)	*P*	OR_per-allele_ b	(95% CI)	*P*	OCAC Studies^c^
*PODXL*	rs1013368	0.38	6,308	2,173	1.00	(0.89–1.10)	0.88	1.02	(0.88–1.18)	0.81	1.01	(0.94–1.08)	0.88	AUS, DOV, GER, HAW, HOP, MAL, POL, SEA, STA, UCI, USC, UKO
*ITGA6*	rs13027811	0.10	8,005	2,660	1.03	(0.92–1.16)	0.57	1.04	(0.67–1.61)	0.87	1.03	(0.93–1.14)	0.57	AUS, DOV, HAW, HOP, MAL, MAY, NCO, NEC, NHS, POL, SEA, UCI, UKO, USC
*MMP3*	rs522616	0.20	8,773	2,985	1.03	(0.94–1.12)	0.58	1.03	(0.84–1.27)	0.74	1.02	(0.96–1.10)	0.55	AUS, DOV, GER, HAW, HOP, MAL, MAY, NCO, NEC, NHS, POL, SEA, STA, UCI, UKO, USC
*PODXL*	rs11768640	0.24	2,952	1,076	0.92	(0.79–1.06)	0.25	0.86	(0.62–1.18)	0.35	0.92	(0.82–1.04)	0.17	AUS, MAL, SEA, UKO, USC
*PODXL*	rs4731799	0.47	2,954	1,077	0.94	(0.80–1.11)	0.48	1.03	(0.85–1.25)	0.75	1.01	(0.92–1.12)	0.82	AUS, MAL, SEA, UKO, USC
*ITGA6*	rs1574028	0.09	2,958	1,080	1.00	(0.83–1.21)	0.98	1.10	(0.54–2.23)	0.79	1.01	(0.85–1.20)	0.89	AUS, MAL, SEA, UKO, USC
***MMP7***	**rs17098236**	**0.08**	**2,945**	**1,074**	**1.14**	**(0.95–1.37)**	**0.17**	**2.34**	**(1.04–5.26)**	**0.04**	**1.19**	**(1.01–1.42)**	**0.04**	**AUS, MAL, SEA, UKO, USC**
*MMP26*	rs11035042	0.11	2,957	1,080	1.04	(0.88–1.24)	0.63	0.63	(0.30–1.31)	0.21	0.99	(0.84–1.16)	0.89	AUS, MAL, SEA, UKO, USC
*FN1*	rs1250229	0.26	2,954	1,075	0.92	(0.79–1.07)	0.26	1.01	(0.76–1.34)	0.96	0.96	(0.86–1.08)	0.50	AUS, MAL, SEA, UKO, USC
*PLOD2*	rs1512900	0.47	2,942	1,070	0.89	(0.76–1.05)	0.16	0.85	(0.69–1.04)	0.11	0.92	(0.83–1.01)	0.09	AUS, MAL, SEA, UKO, USC
*PANX1*	rs1540177	0.40	2,956	1,079	0.94	(0.80–1.10)	0.42	1.18	(0.96–1.45)	0.12	1.06	(0.96–1.17)	0.28	AUS, MAL, SEA, UKO, USC
*PTTG1*	rs17057781	0.14	2,954	1,079	1.04	(0.88–1.22)	0.67	1.32	(0.82–2.15)	0.25	1.07	(0.93–1.23)	0.36	AUS, MAL, SEA, UKO, USC
*CSF1*	rs1999713	0.35	2,957	1,076	0.95	(0.82–1.11)	0.54	1.09	(0.87–1.36)	0.45	1.02	(0.92–1.13)	0.75	AUS, MAL, SEA, UKO, USC
*PTEN*	rs34370136	0.06	2,957	1,079	0.86	(0.68–1.09)	0.22	0.53	(0.12–2.44)	0.42	0.85	(0.68–1.06)	0.15	AUS, MAL, SEA, UKO, USC
*LCN2*	rs3814526	0.04	2,957	1,079	1.20	(0.94–1.52)	0.14	0.38	(0.05–3.12)	0.37	1.14	(0.91–1.44)	0.25	AUS, MAL, SEA, UKO, USC
*TIMP3*	rs5754289	0.17	2,942	1,076	1.02	(0.87–1.19)	0.81	0.93	(0.62–1.41)	0.74	1.00	(0.88–1.14)	0.99	AUS, MAL, SEA, UKO, USC
*DDR2*	rs6693632	0.05	2,954	1,080	0.86	(0.67–1.12)	0.27	0.36	(0.04–2.93)	0.34	0.84	(0.66–1.08)	0.18	AUS, MAL, SEA, UKO, USC
*DDR2*	rs6702820	0.24	2,954	1,080	0.88	(0.76–1.02)	0.09	0.89	(0.64–1.22)	0.46	0.91	(0.80–1.02)	0.10	AUS, MAL, SEA, UKO, USC
*DDR2*	rs10917589	0.07	2,955	1,079	1.00	(0.81–1.24)	0.97	1.62	(0.74–3.56)	0.23	1.05	(0.87–1.27)	0.59	AUS, MAL, SEA, UKO, USC
***TERT***	**rs7726159**	**0.33**	**2,952**	**1,079**	**1.18**	**(1.02–1.37)**	**0.03**	**1.21**	**(0.95–1.53)**	**0.12**	**1.12**	**(1.01–1.25)**	**0.03**	**AUS, MAL, SEA, UKO, USC**

^**a**^MAF in controls.

^**b**^ORs, 95% CI and p-values are adjusted for age (at interview in controls, at diagnosis in cases) and study site.

^**c**^OCAC studies not listed for *PODXL* rs1013368, *ITGA6* rs13027811 and *MMP3* rs522616 were excluded from analysis because of QC failures.

**Figure 1:**
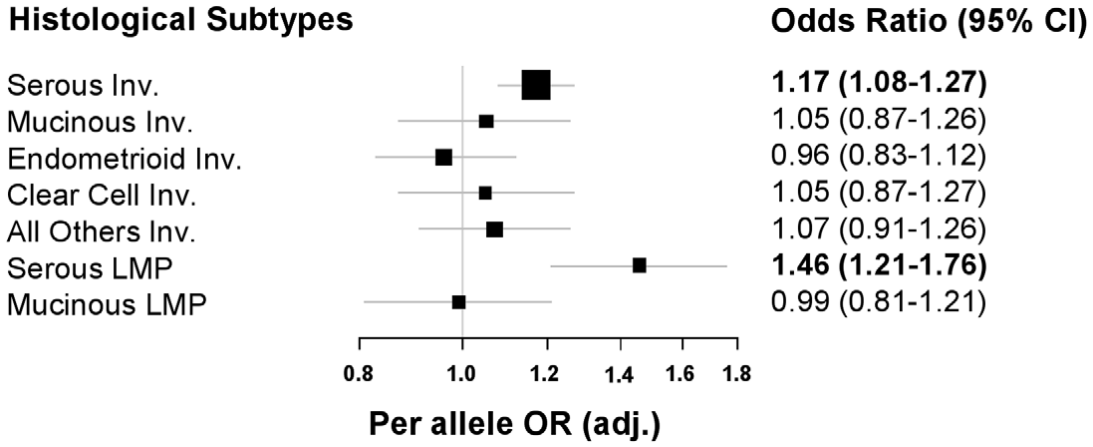
Histology-specific adjusted per allele risk estimates for rs7726159 for all ethnicities. Lines indicate 95% confidence intervals; bolded ORs and 95% CIs indicate statistically significant estimates (*P*<0.05); size of the solid box is the proportionate sample size for each histology sub-group with genotype data.

However, adjusted log additive estimates for *TERT* (telomerase reverse transcriptase) rs7726159 retained a statistically significant *p*-value in the replication study of non-Hispanic White serous invasive cases and controls (*P*_per-allele_ = 0.03), and showed evidence of log additive effects across genotypes. We re-analysed this SNP combining discovery and replication data and observed some evidence of between-study heterogeneity (*p* = 0.027) which again improved with the exclusion of the smaller studies (USC and MAY; *p* = 0.16). Risk estimates for serous invasive ovarian cancer adjusted for age and study site remained statistically significant in the combined dataset [adj. OR_per-allele_ 1.14 (1.04–1.24) *p* = 0.003; Table 3]. Likewise, in exploratory analyses of genotype data on all ethnicities stratified by histological subtype, a increased risk associated with this SNP was observed for serous invasive cases in models adjusted for age, site and ethnicity [adj. OR_per-allele_ 1.17 (1.08–1.27) *p* = 7.21×10^−5^]. *TERT* rs7726159 was also associated with serous borderline tumors, but not with any other invasive or borderline subtypes (Table 4, and Figure 1). For *MMP7* rs17098236, the combined age- and site-adjusted estimate from the log additive model suggested an association with serous ovarian cancer but the point estimates were not in the same direction as those obtained in discovery analysis (0.84 vs.1.19; see Table S3 and Table 2). All other SNPs in the smaller replication study failed to replicate the significant associations observed in the discovery sample.

**Table 3:** Combined discovery and replication analysis: site-specific and combined risk estimates for serous ovarian cancer for *TERT* rs7726159 among non-Hispanic whites.

			Heterozygotes			Homozygotes			Per-allele		
Study	Controls	Serous Cases	aOR	(95% CI)	*P*	aOR	(95% CI)	*P*	aOR	(95% CI)	*P*
SEA	1,213	383	**1.35**	**(1.05–1.73)**	**0.019**	**1.65**	**(1.14–2.38)**	**0.008**	**1.30**	**(1.10–1.54)**	**0.003**
AUS	1,202	636	**1.30**	**(1.06–1.60)**	**0.012**	1.27	(0.92–1.76)	0.148	**1.18**	**(1.02–1.37)**	**0.025**
MAL	764	264	**1.50**	**(1.10–2.03)**	**0.009**	1.37	(0.86–2.19)	0.184	**1.27**	**(1.03–1.57)**	**0.025**
UKO	564	235	0.95	(0.68–1.32)	0.754	0.89	(0.50–1.59)	0.685	0.95	(0.74–1.21)	0.658
USC	218	128	0.71	(0.45–1.13)	0.152	0.53	(0.24–1.15)	0.108	0.72	(0.51–1.01)	0.056
MAY	61	125	1.53	(0.80–2.94)	0.198	0.80	(0.26–2.40)	0.684	1.09	(0.67–1.78)	0.732
**Combined (all studies)**	4,022	1,771	**1.23**	**(1.09–1.39)**	**0.001**	1.19	(0.98–1.44)	0.072	**1.14**	**(1.04–1.24)**	**0.003**

^**a**^Estimates are adjusted for age (at interview in controls, at diagnosis in cases) and additionally for study site in combined (all studies) estimates.

**Table 4:** Combined discovery and replication analysis: risk estimates for *TERT* rs7726159 for all races according to tumor behaviour and histological subtypes.

				Heterozygotes			Homozygotes			Per-Allele		
Tumor Behavior	Histological Subtype	aControls	aCases	bOR	(95% CI)	*P*	bOR	(95% CI)	*P*	bOR	(95% CI)	*P*
**Invasive**	Serous	4138	2196	**1.30**	**(1.16–1.45)**	**5.7×10^−6^**	**1.25**	**(1.05–1.49)**	**0.011**	**1.17**	**(1.08–1.27)**	**7.21×10^−5^**
	Mucinous	4138	271	1.15	(0.88–1.49)	0.31	1.01	(0.66–1.54)	0.98	1.05	(0.87–1.26)	0.63
	Endometrioid	4138	454	0.81	(0.65–0.99)	0.045	1.09	(0.80–1.48)	0.57	0.96	(0.83–1.12)	0.64
	Clear Cell	4138	261	1.03	(0.79–1.35)	0.83	1.12	(0.75–1.69)	0.57	1.05	(0.87–1.27)	0.60
	All others	4138	355	1.08	(0.86–1.37)	0.50	1.49	(0.80–1.64)	0.45	1.07	(0.91–1.26)	0.38
**Borderline/LMP**	Serous	4138	251	**1.63**	**(1.21–2.18)**	**0.001**	**2.04**	**(1.38–3.02)**	**0.0004**	**1.46**	**(1.21–1.76)**	**6.63×10^−5^**
	Mucinous	4138	249	1.13	(0.85–1.49)	0.40	0.85	(0.53–1.36)	0.51	0.99	(0.81–1.21)	0.92

^**a**^Cases and controls derived from AUS, MAL, MAY, SEA, UKO and USC studies.

^**b**^Estimates are adjusted for age (at interview in controls, at diagnosis in cases), race and study site.

## Discussion

Herein we report a large-scale analysis of 1,309 SNPs in 173 genes selected for their putative role in stromal epithelial cross talk, using a two-stage design for assessment of ovarian cancer risk. In the discovery stage we used data from two OCAC case-control studies (AUS and MAY) of predominantly non-Hispanic White women, and observed that SNPs in several genes were associated with risk of serous tumours in unadjusted log-additive models (Table S3). The most significant associations observed (*PODXL* rs1013368, *ITGA6* rs13027811, and *MMP3* rs522616; *P*_Trend_≤0.001; Table 1) were then genotyped in a total of sixteen OCAC studies including additional samples from discovery studies (AUS and MAY), and also from non-serous histologies and all ethnicities. None of these three SNPs were significantly associated with ovarian cancer risk (*P*_per-allele_≥0.5). The power of the replication sample to detect the odds ratios observed in the discovery set at a type 1 error rate of 0.05 assuming log additive effects was >99.9% for all three SNPs. Combining discovery and replication data would have provided greater power to detect a significant effect [18], but this was not considered for these SNPs because estimates were unequivocally null in replication analysis and/or in the opposite direction compared to the smaller discovery dataset.

We analysed an additional 18 SNPs, including one in *FGF2* reported elsewhere [17] in a second smaller replication study using five case-control studies from OCAC, and found evidence of an allelic association between *TERT* rs7726159 and serous tumors (Table 2). Although the PPV for *TERT* rs7726159 was 1.4%, it was not selected for the larger replication stage in all sixteen OCAC case-control studies because of limited resources. Our estimate from the replication study, adjusted for age and study site, showed an overall 12% increased risk of serous ovarian cancer associated with each minor allele among non-Hispanic Whites. Site-specific estimates were also statistically significant in case-control studies with the largest samples sizes (SEA, AUS and MAL) (Table 3). We detected significant study heterogeneity in this combined sample of all studies (*p* = 0.027), and this effect was attenuated when the smallest sample sizes (USC and MAY) were removed from the dataset; *p* = 0.16). Inclusion of data on all ethnicities additionally adjusted for race resulted in a significance level (adj. *P*_per-allele_ = 7.21×10^−5^) that met the conservative Bonferroni adjustment for multiple testing (0.05/21 = adj. *P*_per-allele_≤0.0024). In addition, the estimates from log-additive models for *TERT* rs7726159 in the combined discovery and replication non-Hispanic White samples would almost meet Bonferroni adjustment (adj. *P*_per-allele_ = 0.003).

*TERT* encodes the catalytic subunit of telomerase and activation of telomerase has been implicated in human cell immortalization and cancer cell pathogenesis. *TERT* was selected as a candidate gene because it serves as an epithelial stem cell marker [19] and we hypothesized that cross-talk modifies critical aspects of epithelial transformation. *TERT* is a ribonucleoprotein enzyme that maintains telomere ends, and is essential for the replication of chromosomes and suppression of cell senescence. Telomere dysfunction is associated with genomic instability and consequently increased risk of tumor formation [20]. The rs7726159 variant resides in intron 3 of *TERT* and has no obvious functional significance, but it could be in linkage disequilibrium with another functional or causal SNP within the gene. An alternative explanation for the observed association is population stratification, which occurs when allele frequencies differ with population subgroups, or when cases and controls are drawn from different subgroups. We suggest that this is not a likely explanation because cases and controls were drawn from the same source populations within each study, and replication analyses were restricted to non-Hispanic White women or adjusted for ethnicity where applicable. However, it is possible that the association with serous ovarian cancer may vary across populations because of interaction with other genes or environmental factors, and additional studies would be required to confirm these findings.

**Figure 2:**
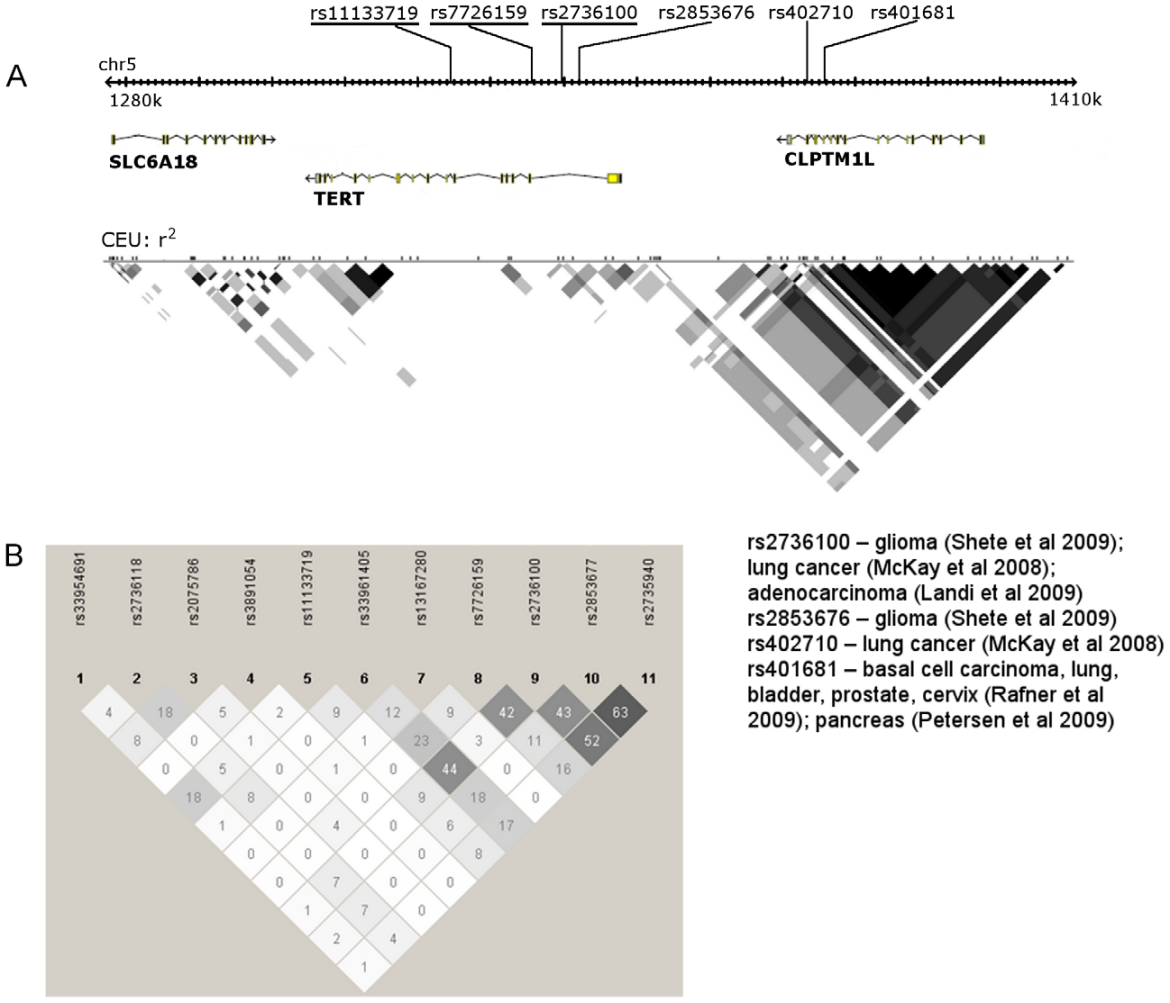
Gene map and LD plot of *TERT-CLPTM1L* locus and associated SNPs. Colour scheme is based on r^2^ values in Haploview; white r^2^ = 0; shades of grey 0<r^2^<1; black r^2^ = 1. Block definition is based on the method of Gabriel et al [54]. (A) Gene map of SNPs genotyped in the discovery stage (underlined) relative to other *TERT* SNPs associated with cancer phenotypes (inset) and LD plot based on HapMap CEU samples. (B) Haploview plot of all *TERT* SNPs genotyped in 1,047 non-Hispanic White controls in our study; numbers in squares are pairwise r^2^ values between SNPs.

Although *TERT* variants have not been previously reported to be associated with ovarian cancer, a recent meta-analysis of two GWAS identified another SNP in *TERT*, rs2736100, as significantly associated with gliomas (OR = 1.27; *P* = 1.50×10^−17^) [21]. GWAS have found that rs2736100 is also associated with lung cancer (OR = 1.14; *P* = 4×10^−6^) [22] and more specifically, with the adenocarcinoma subtype (OR = 1.23; *P* = 3.02×10^−7^) [23] (Figure 2A). Associations have also been reported between the *TERT- CLPTM1L* (cleft lip and palate transmembrane 1-like gene - cisplatin resistance-related protein 9-) locus and lung cancer (rs402710; OR = 1.17; *P* = 2×10^−7^) [22], basal cell carcinoma (rs401681; OR = 1.20; *P* = 4.8×10^−9^) [24], pancreatic cancer (rs401681; OR 1.19; (*P* = 3.66×10^−7^) [25], and multiple cancer types that are known to originate in the epithelium, including bladder, prostate and cervical cancer [26]. We genotyped rs2736100 in the discovery samples and found a borderline, but inverse, association with serous ovarian cancer [OR = 0.88 (0.77–1.01) *P*_Trend_ = 0.06]. We also found a borderline association with rs11133719 and serous ovarian cancer risk [OR = 0.81 (0.67–0.98) *P*_Trend_ = 0.025] in discovery samples. Linkage disequilibrium (LD) estimation between the 11 *TERT* SNPs that we genotyped in stage 1 in 1,047 non-Hispanic White controls showed a moderate pairwise correlation between rs2736100 and rs7726159 (r^2^ = 0.43; Figure 2B) but rs7726159, which we selected from NIEHS, is not in HapMap and so has not been genotyped in GWAS of ovarian or other cancers. Further analysis of this locus is necessary in order to definitively identify the causal SNP(s) at this locus.

To our knowledge, this is the first comprehensive evaluation of genes involved in stromal epithelial cross-talk and serous ovarian cancer. Candidate gene and SNP selection for discovery stage analysis was aimed at optimizing the likelihood of detecting a signal by including tagging and putatively functional SNPs with minor allele frequency (MAF)>5%. Although a tagSNP approach has been shown to improve the power of the study for common variants [27], modest effects from SNPs with low MAFs may remain undetected. This was illustrated in a recent re-analysis of two SNPs in the *DCN* gene that failed to achieve the minimal *P*_Trend_≤0.05 in stage 1 analysis, but conferred a small but significantly decreased risk of serous ovarian cancer in a combined analysis of data from two additional studies [28]. We therefore suggest caution in interpreting null findings, and the need for large discovery and replication studies. Our discovery study was reasonably well powered, so the failure to find any associations with SNPs in genes involved in stromal epithelial cross-talk, except in *DCN* and *TERT*, suggests that genetic variation in this pathway is not a major determinant of serous ovarian cancer risk.

In summary, we have identified an association between *TERT* rs7726159 and serous ovarian cancer in a large sample of non-Hispanic White women participating in five OCAC case-control studies. We plan to further our investigation of this SNP and others in linkage disequilibrium with it, to determine whether *TERT*, *CLPTM1L* or another gene in the region is the functional target of this association. Our study adds to the growing evidence that, as well as the 8q24 locus [21], [29], [30]–[32], the *TERT-CLPTM1L* locus at 5p15.33, is a general cancer susceptibility locus. This is particularly interesting given the key roles of c-*MYC* (the nearest gene to the 8q24 locus) and *TERT* in tumorigenesis. *TERT* and *MYC* are both expressed in normal and transformed proliferating cells, and can induce immortalization when constitutively expressed [33]. The *TERT* promoter contains numerous MYC binding sites that mediate *TERT* transcriptional activation [34], suggesting that *TERT* is a target of MYC activity. Although *TERT* variants have not been previously reported to be associated with ovarian cancer, multiple genome-wide association studies have reported associations with this locus and risk of other cancers. Further analyses of this locus, including fine mapping, resequencing and functional assays, will be necessary to definitively identify the causal SNP(s).

## Materials and Methods

### Study populations

Approval from respective human research ethics committees was obtained, and all participants provided written informed consent. Sixteen OCAC case-control studies (summarized in Table S2) contributed data to this two-stage risk analysis. Samples in the discovery stage were derived from two case-control studies, AUS (550 cases and 1,101 controls) and MAY (125 cases and 61 controls). Cases in the discovery set were all diagnosed with serous carcinoma of the ovary, fallopian tube or peritoneum, and most of the participants were non-Hispanic white women. Cases and controls from an additional 14 OCAC studies, as well as an additional 284 AUS and 477 MAY samples, including cases with other histologies, were included in a stage 2 analysis designed to replicate the most promising SNPs from the discovery stage. Fifteen studies used population-based case and control ascertainment, and one (MAY) was clinic-based. All studies have been previously described [7], [35], [36]. The final combined dataset of all discovery and replication samples consisted of a total of 10,067 controls (9,953 were self-classified as non-Hispanic White) and 5,976 ovarian cancer cases of all histologies and morphologies, including 3,734 serous invasive cases (3,710 were self-classified as non-Hispanic Whites) (Table S2).

### Candidate gene and SNP selection

Our approach and our choice of candidate genes was based on extensive preliminary data we have accumulated from gene expression profiles of co-cultured of theca fibroblast and epithelial ovarian cells (I. Haviv, personal communication), and expression profiles of murine ovarian epithelial cells identifying candidates that are regulated through the estrus cycle [37], [38] (see Text S1). A compiled list of candidates was uploaded on the Ingenuity Pathway Analysis web interface and GeneSpring GX in order to obtain further candidates inferred from the literature. Prioritisation based on literature evidence for a plausible role in oncogenesis resulted in a list of 255 candidate genes of interest including *CXCL9*, *CTGF*, *LCN2*, *DCN*, and *VIL2*. CXCL9 is associated with ovarian cancer survival and acts by recruiting T-cells and inducing immune surveillance [39], and is expressed in epithelial cells co-cultured with fibroblasts. CTGF is likely to be the driver of the CAF phenotype. CTGF (TGFβ-stimulated) expression is associated with desmoplastic stroma [40] and elevated angiogenesis [41]. *LCN2*, *DCN* and *VIL2* were regulated through the murine estrus cycle, and appear to be hormone responsive (either directly or indirectly) [37]. Furthermore, comparison with expression profiles of human ovarian carcinomas [42], [43] showed that all three are differentially expressed in tumors compared with normal epithelial cells. Further details for candidate gene selection and justification are provided in Text S1 and Table S1.

We identified SNPs within 5 kb of these 255 genes (58,114 SNPs in total from dbSNP, Ensembl, the International HapMap Consortium [44], Perlegen Sciences [45], SeattleSNPs [pga.mbt.washington.edu/], NIEHS SNPs [http://egp.gs.washington.edu], and the Innate Immunity PGA [http://www.nhlbi.nih.gov/resources/pga/]. We used the binning algorithm of ldSelect [46] to identify 4,567 tagSNPs among these (r^2^>0.8) and minor allele frequencies (MAFs)>0.05 based on the most informative available source (84% of genes used HapMap, 10% used SeattleSNPs, 3% used Perlegen Sciences, 2% used NIEHS SNPs, and 1% used Innate Immunity PGA). We prioritized the list to 166 genes based on known function and the number of bins in each gene (excluding genes with a large number of bins), in an attempt to identify ∼1,500 key SNPs. Based on Illumina design scores, we picked the best tagSNP in each bin (or two tagSNPs, if there were >10 tagSNPs in a bin but none of them had an optimal design score). We also used PATROCLES (www.patrocles.org,) to identify supplemental SNPs with MAFs>0.05 in microRNA binding sites or non-synonymous SNPs from public databases to the potential SNP list. This identified an additional 170 miRNA binding site SNPs and nsSNPs with Illumina design scores>0.6. In total this gave 1,410 tagSNPs, miRNA binding site SNPs and nsSNPs. In order to reach the final total of 1,536 SNPs for the Illumina GoldenGate assay, we added tagSNPs in another 12 candidate genes with MAF≥0.01. The final list of 1,536 SNPs included 106 supplemental SNPs and 1,430 tagSNPs in 173 genes (see Table S1).

### Genotyping and quality control

The discovery samples were predominantly non-Hispanic White women with serous ovarian cancer and controls derived from two studies, the AUS and MAY studies, and were genotyped using the Illumina GoldenGate assay and Illumina BeadStudio software [47], [48]. Plates were prepared containing randomly mixed cases and controls, with two duplicated samples and one blank per plate (n = 20). The Illumina GoldenGate assay was performed according to the manufacturer's instructions. Following completion of the assay, all plates were analysed using Illumina BeadStudio software version 3.1.0.0. The original raw genotype dataset contained genotype information for 1,920 samples (including blanks and duplicates) and 1,536 SNPs. Following automatic clustering, SNPs were ranked using their GenTrain score (ranging from 0 to 1) and those with GenTrain scores<0.5 were manually checked and adjusted according to Illumina guidelines. Samples with call rates below 95% and SNPs with call rates below 98% were excluded. A total of 1,292 SNPs passed this initial quality control (QC). Genotyping quality was also assessed using tests for Hardy-Weinberg equilibrium (HWE). Plots were examined for SNPs with significant deviations from HWE in controls (0.001<*P*<0.05) and the genotype data was excluded if the clustering was found to be suboptimal. SNPs with *P*_HWE_<0.001 were excluded from analysis. In addition, we genotyped 17 SNPs in *CXCL9*, *CTGF*, *LCN2*, *DCN*, and *VIL2*, that had not been amenable to the Illumina GoldenGate assay or failed QC criteria, at the Queensland Institute of Medical Research using MALDI-TOF mass spectrophotometric mass determination of allele-specific primer extension products with Sequenom's MassARRAY platform and iPLEX Gold technology. The final discovery dataset for analysis consisted of 675 cases and 1,162 controls with genotype data on 1,309 SNPs.

The three SNPs in *PODXL*, *ITGA6* and *MMP3* selected for replication by all participating OCAC sites (with the exception of *MMP3* at the MAY site) were genotyped with the TaqMan allele discrimination assay (Taqman Applied Biosystems, Foster City, CA), using primers designed by Assays-by-Design (Applied Biosystems). MAY genotyping of *MMP3* rs522616 was performed as part of a 1,536 Illumina Golden Gate Assay at the Mayo Clinic with cases and controls randomly mixed within each plate. Additional genotyping details are provided elsewhere [49].

Samples from five OCAC case-control studies (MAL, SEA, UKO, USC and additional samples from AUS) were genotyped for these and other replication SNPs, at the Queensland Institute of Medical Research using Sequenom iPLEX Gold technology. Primer design was carried out according Sequenom's guidelines using MassARRAY Assay Design software (version 1.0). Multiplex PCR amplification of fragments containing target SNPs was performed using Qiagen HotStart Taq Polymerase and a Perkin Elmer GeneAmp 2400 thermal cycler with 10 ng genomic DNA in 384 well plates. Shrimp Alkaline Phosphatase and allele-specific primer extension reactions were carried out according to manufacturer's instructions for iPLEX GOLD chemistry. Assay data were analysed using Sequenom TYPER software (Version 3.4).

Only replication SNPs that met OCAC's QC criteria (including >95% call rate, and >98% concordance between duplicates) were included in the analysis [50].

### Statistical analysis

The primary test for association in stage 1 was univariate analyses of the relationship between SNP genotypes and risk of serous ovarian cancer using the PLINK v0.99 Whole Genome Association Analysis toolset (http://pngu.mgh.harvard.edu/purcell/plink/) [51]. Single-marker basic allelic association (χ^2^ 1df) tests (–*assoc* option) analyses were performed on each of the 1,309 post-QC SNPs in a total of 1,837 women. PLINK default thresholds were utilized, resulting in further exclusions: maximum missing genotypes per person≤0.10 (–*mind* option), maximum failed genotypes per SNP≤0.10 (–*geno* option), MAF≥0.01 (–*maf* option). Summary statistics were obtained for each SNP on the frequency of missing genotype data among cases and controls as well as a comparison of ‘missingness’ between cases and controls using the Fisher's exact test (–*test-missing* option). Deviations from expected HWE proportions were analysed using the Fisher's exact test and the MAFs were also estimated for all SNPs. The Cochran Armitage Trend test (χ^2^ 1df) assuming the log additive model (–*model* option) was performed to test the association between the minor allele of each SNP and serous ovarian tumors.

Selection of stage 1 SNPs for replication analyses in stage 2 was prioritized as follows: first, SNPs with at least one failed duplicate, SNPs with a significantly different proportion of missing genotype data between cases and controls (*P*_Miss_<0.05), SNPs not conforming to HWE criteria (see Genotyping and quality control) for either cases, controls or both, and SNPs with no significant trend in allelic dose response (*P*_Trend_>0.05) were excluded; secondly, we estimated from the remaining SNPs which were likely to be the best predictors of serous ovarian cancer risk by calculating the positive predictive value (PPV) using the *P*_Trend_ values, the power of the study to detect this association, and the prior probability of 0.0001 [52]. Cases and controls from up to 14 additional studies participating in OCAC were included in replication analyses. We selected the three SNPs with the highest PPV for the larger replication analysis by all studies. Some additional individuals from AUS and MAY (not in the discovery set) were included in the replication analysis. Replication samples were examined to determine the distribution of race/ethnicity across studies, and analyses were restricted to White non-Hispanic women with serous invasive ovarian tumors. Significant differences by study site between age at interview for controls and age and diagnosis for cases were assessed using the Student's *t*-test for comparison of means. The MAF for each SNP was estimated from the control population for each study. The combined odds ratios (OR) and their 95% confidence intervals (95% CIs) were obtained from unconditional logistic regression models for each SNP genotype. Assuming a log additive model of inheritance, the per-allele ORs and their 95% CIs associated with serous invasive ovarian cancer in non-Hispanic Whites for each SNP selected for replication were estimated by fitting the number of rare alleles carried as a continuous covariate. Separate comparisons for women with one copy (heterozygotes) and women with two copies (rare homozygotes) of the minor allele vs. those with no copies (reference homozygotes) were conducted for all replication SNPs. Between-study heterogeneity was assessed using the likelihood ratio test to compare logistic regression models with and without a genotype-by-study interaction term. Risk estimates from all replication analyses were adjusted for age at diagnosis for cases or age at interview for controls and study site. Exploratory analyses combining all ethnicities were additionally adjusted for ethnicity. Forest plots generated in exploratory analyses according to histological subtype were obtained using the *rmeta* library (v2.15) implemented in the R project for Statistical Computing (http://www.r-project.org/), and LD plots were generated using Haploview v4.1 [53]. All tests for association were two-tailed, and unless otherwise specified, statistical significance was assessed at *p*<0.05 and tests for association in stage 2 were performed in STATA v. 9.0 (StataCorp, USA).

## Supporting Information

Figure S1 Study design for two-stage analysis of selected SNPs in genes involved in stromal-epithelial interactions in the Ovarian Cancer Association Consortium (OCAC).
(0.08 MB TIF)

Table S1 Candidate genes, putative role/special justification for selection and reference list.
(0.05 MB DOC)

Table S2 Characteristics of serous ovarian cancer cases and controls used in discovery and replication analyses according to contributing OCAC study.
(0.05 MB DOC)

Table S3 SNPs successfully genotyped (Illumina & Sequenom) in the discovery stage with P_Trend_≤0.05 for serous ovarian cancer risk.
(0.12 MB DOC)

Table S4 Study heterogeneity *p*-values for serous ovarian cancer risk estimates among non-Hispanic whites for SNPs reported in Table 2.
(0.04 MB DOC)

Text S1 Candidate gene selection and justification.
(0.06 MB DOC)
