# Supplementary figures and images for: Evaluation of Candidate Stromal Epithelial Cross-Talk Genes Identifies Association between Risk of Serous Ovarian Cancer and TERT, a Cancer Susceptibility “Hot-Spot”
Source: PLoS Genet. 2010 Jul 8;6(7):e1001016. doi: 10.1371/journal.pgen.1001016 (PMC2900295; doi:10.1371/journal.pgen.1001016)

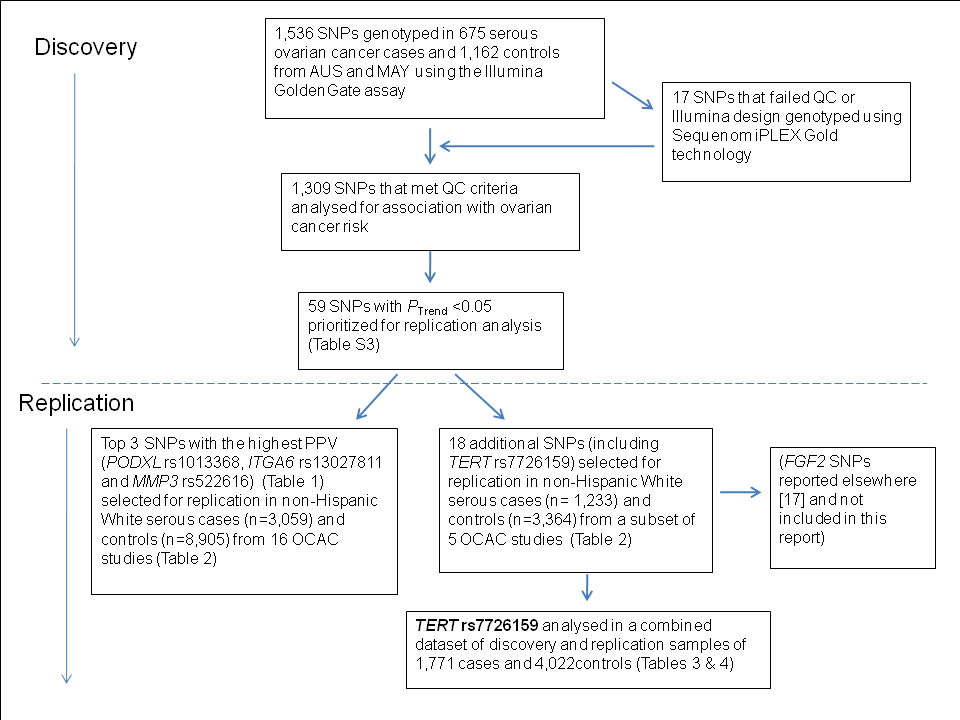

Supplement: Figure S1 — Study design for two-stage analysis of selected SNPs in genes involved in stromal-epithelial interactions in the Ovarian Cancer Association Consortium (OCAC). (0.08 MB TIF) [file pgen.1001016.s001.tif]
