# Supplementary material for: Evaluation of Candidate Stromal Epithelial Cross-Talk Genes Identifies Association between Risk of Serous Ovarian Cancer and TERT, a Cancer Susceptibility “Hot-Spot”
Source: PLoS Genet. 2010 Jul 8;6(7):e1001016. doi: 10.1371/journal.pgen.1001016 (PMC2900295; doi:10.1371/journal.pgen.1001016)
Supplement: Table S1 — Candidate genes, putative role/special justification for selection and reference list. (0.05 MB DOC) [file pgen.1001016.s002.doc]

**Table S1: Candidate genes, putative role/special justification for selection and reference list**

| **Functional Role** | **Gene(s)** | ***References** |
| --- | --- | --- |
| Antagonizes metastasis promotion by TGFb | *BMP1, BMP4* | [39,50] |
| AP1/TRE sequence over-represented in promoters of co-culture responsive genes (our met-analysis) | *FOS, JUN* | [78] |
| At the cross-road of WNT-dependent signalling | *DKK1, WISP1, WNT10B, WNT2, WNT5A* | †Haviv et al, [66] [12] [64] |
| Cyr61 at the cross-road of TGFb and cAMP-dependent signalling in invasiveness | *CYR61* | [12], [73] |
| Degradation of ECM and basement membrane would remove the insulator between epithelium and stroma, and increase wound healing and co-culture reaction | *MEST, MFAP4,MMP1, MMP14, MMP2, MMP26, MMP3, MMP7, MMP9, MPI, P4HA2, PLAT, PLAU, PLAUR, PLOD, PLOD2, SELENBP1, SERPINB2, SERPINB7, SERPINE1, SERPING1, TIMP1, TIMP3* | †Haviv et al, [12,49,64-66,72,74] |
| EGF-ligands are provided by stroma, either connective tissue or macrophages | *AREG, EGF, ERBB3* | [76,77] |
| EMT-driver | *TWIST1* | †Haviv et al |
| Establishes an intercellular signalling pathway that plays a key role in regulating stem cell and apoptosis | *NOTCH3* | †Haviv et al |
| Gap junctions regulate cancer-stromal interaction | *GJB1, GJB2, PANX1* | [12,68,69] |
| Implicated in co-culture-like reaction in vivo through correlation with overall co-culture signature in tumor tissue expression profiles | *ADAM8, CTSK, GPX4, HOXB2, S100A7, SAT, SIAT9, TYROBP* | [39] †Haviv et al |
| Inflammation | *C3, CCL11, CCL13, CCL7, cig5, CXCL1, CXCL14, CXCL3, CXCL6, CXCL9, CXCR6, IL1B, IL1R1, NFKB2, NFKBIA, PTGES, PTGS1, PTGS2, SSA1, T1A-2, TNF, TNFAIP2, TNFAIP3, TNFAIP6, TNFRSF12A, TNFRSF1B, TNFSF10, TNFSF7, TNFSF9* | [12,49,64-66,72] [43,44,46,75,84] †Haviv et al |
| Interferon | *G1P2, G1P3, GAS1, IFI16, IFI35, IFIT1, IFITM1, IRF7, MX1, OAS1, OAS3, OSMR, PRKR, PRKRA, PRKRIR, STAT1, STAT3* | [43,64,65] [12,39,44] †Haviv et al, |
| Key promoter of connective tissue and cancer stroma | *CTGF, FGF2, FST, PDGFB, PDGFRB, TGFB2, TGFB3, TIEG* | [12,44,49,64,72] |
| Major promoter of tumor associated macrophages | *CSF1* | [77] |
| One of the six milestone changes in cancer cells and suggested etiology of cancer stroma; angiogenesis | *THBS4* | †Haviv et al |
| One of the six milestone changes in cancer cells and suggested etiology of cancer stroma; senescence | *TERT* | [71] |
| One of the six milestone changes in cancer cells; angiogenesis | *HIF1A, VEGF* | [14,15,64,65,77] |
| One of the six milestone changes in cancer cells; cell cycle, cancer-stromal interactions, and apoptosis | *BUB1, CCND2, MCM2, MCM6, MKI67, PTTG1, CYC1* | [64-66,70] †Haviv et al |
| One of the six milestone changes in cancer cells; evasion of apoptosis | *BCL2L1* | [64,65] [12] |
| One of the six milestone changes in cancer cells; lymphangiogenesis | *VEGFC* | †Haviv et al |
| One of the six milestone changes in cancer cells; stem cell | *CD24, CD44* | [61] |
| One of the six milestone changes in cancer cells; survival | *IGFBP3, IGFBP4, IGFBP5, PTEN* | [12,64-66] |
| p53-responsive gene, implicated in cancer stroma, chosen based on correlation with overall co-culture response | *PODXL* | †Haviv et al |
| Tissue remodelling ECM component | *FN1* | [12] |
| Tyrosine kinase that is activated by physical distortion of cell shape, often responds to ECM density, which is in turn controlled by tissue remodelling stroma | *DDR2* | †Haviv et al |
| Ovulation-related gene regulation *in vivo* and aberrant expression in ovarian carcinomas | *C3, DCN, GATA6, IGFBP4, LCN2, TACSTD1, VIL2,* | [12,65,67,79,80-83] |
| Other | *ANKRD1, BPNT1, BST2, CFLAR, CRLF3, DAB2, ,DLG7, DPP4, DSC3, DUSP5, EGR2, EIF4EBP2, FLT3LG, GABARAPL1, H1F0, ID2, IFITM2, IL6, IL6ST, IL8, INHBA, ITGA6, ITGAV, ITGB1, LAMC1, MAPK1, NT5E, OGT, PTP4A1, PTPN1, RGS2, SNAI1, SORD, SORT1, SOX9, SPARC, SPP1, SPRY1, STEAP, TYK2, VDR, ZNF354A* | [64-66] [12,14,44,45,49,50,70,72,78,85] |

*See Text S1

†Haviv I, Boussioutas A, Thorne N, Holloway A Robbie, MJ, Speed T and Bowtell DDI. The transcriptional response to stromal invasion by ovarian epithelium: A model for tumor microenvironment (manuscript in preparation)
