# Supplementary material for: Evaluation of Candidate Stromal Epithelial Cross-Talk Genes Identifies Association between Risk of Serous Ovarian Cancer and TERT, a Cancer Susceptibility “Hot-Spot”
Source: PLoS Genet. 2010 Jul 8;6(7):e1001016. doi: 10.1371/journal.pgen.1001016 (PMC2900295; doi:10.1371/journal.pgen.1001016)
Supplement: Table S2 — Characteristics of serous ovarian cancer cases and controls used in discovery and replication analyses according to contributing OCAC study. (0.05 MB DOC) [file pgen.1001016.s003.doc]

Table S2: Characteristics of serous ovarian cancer cases and controls used in discovery and replication analyses according to contributing OCAC study.

| **Study**  **(Location)** | **Case Ascertainment** | **Serous Cases^a^** |  |  | **Control Ascertainment** | **Controls^a^** |  |  |
| --- | --- | --- | --- | --- | --- | --- | --- | --- |
|  |  | **Total (N)** | **Age^b^ (Mean** | **±SD)** |  | **Total** | **Age^b^ (Mean** | **±SD)** |
| AUS  (Australia) | Surgical treatment centres throughout Australia, and cancer registries of Queensland, South and Western Australia, New South Wales and Victoria; | 634 | 60.1 | ± 10.4 | Population based: Randomly selected from Commonwealth Electoral roll | 1,301 | 57.0 | ± 11.8 |
| DOV  (Washington, USA) | Cases ascertained from a 13-county area in western Washington state | 303 | 58.9 | ± 8.6 | Population based: Random digit dialling. Frequency matched to cases for race/ethnicity and 5-year age groups | 716 | 55.6 | ± 9.7 |
| GER  (Germany) | Cases ascertained through all treating hospitals of the study area | 107 | 58.1 | ± 10.0 | Population-based: identified through population registries and matched to cases by age and study area | 433 | 55.4 | ± 11.8 |
| HAW  (Hawaii, USA) | Rapid cases ascertainment from Hawaii Tumour Registry | 36 | *56.1 | ± 9.8 | Randomly selected from Hawaii Department of Health Annual Survey of representative households | 158 | *57.3 | ± 11.3 |
| HOP  (NY, OH and PA, USA) | Registries, physician offices, pathology databases, hospital records | 159 | 60.0 | ± 11.5 | Population based: Random digit dialling | 603 | 57.3 | ± 10.2 |
| MAL  (Demark) | Incident cases (35-79 years of age) from Copenhagen and Fredriksberg and surrounding counties | 273 | 60.0 | ± 10.6 | Population based: random sample of female population in areas cases selected from | 798 | 57.3 | ± 11.2 |
| MAY  (Mayo Clinic, USA) | Cases attending the Mayo Clinic from six surrounding states | 221 | 62.1 | ± 12.0 | Clinic based: Women seeking general exams at Mayo Clinic | 442 | 60.2 | ± 13.1 |
| NCO  (NC, USA) | Identified from 48 counties in the state by rapid-case ascertainment | 366 | 58.4 | ± 9.7 | Controls identified from same counties as cases and frequency matched to cases for age and race | 748 | 55.1 | ± 11.9 |
| NEC  (New England, USA) | Identified through hospital tumour boards and state cancer registries in New Hampshire and Massachusetts | 269 | 55.5 | ± 11.3 | Controls identified through random digit dialing, townbooks and drivers’ license lists; frequency matched to cases for age and race | 928 | 51.1 | ± 13.1 |
| NHS  (Nurses’ Health Study, USA) | Self-report on biennial questionnaires or via the National Death Index; confirmed by pathologist review | 59 | *64.9 | ± 7.5 | Nested-case control study design, with three controls matched per case on age at blood collection. | 362 | *64.6 | ± 7.8 |
| POL  (Poland) | Cases recruited through a rapid identification system at participating hospitals and Cancer Registries | 114 | *55.3 | ± 10.9 | Controls randomly selected through Polish Electronic System demographic database | 606 | *56.1 | ± 10.8 |
| SEA  (UK) | Cases <70 years from East Anglian, West Midlands & Trent regions of England. Prevalent cases diagnosed 1991-1998; incident cases from 1998 onwards | 367 | 56.6 | ± 9.5 | Selected from the EPIC-Norfolk cohort of 25,000 individuals aged 45-74 based in the same geographical region as cases | 1,213 | 54.9 | ± 9.1 |
| STA  (CA, USA) | Population- and family-based | 169 | 52.5 | ± 8.7 | Population- and family-based | 181 | 48.1 | ± 10.2 |
| UCI  (Orange and San Diego Counties, USA) | Case recruitment by rapid ascertainment through Cancer Surveillance Program in area surrounding UCS | 148 | 60.5 | ± 12.0 | Controls identified through random digit dialling, frequency matched to cases by age group and race/ethnicity | 425 | 55.5 | ± 12.0 |
| UKO  (UK) | Incident cases from 10 gynaecological oncology NHS centres throughout the UK | 235 | 61.3 | ± 10.3 | Postmenopausal women from the general population participating in the UK Collaborative Trial of Ovarian cancer Screening | 566 | 65.0 | ± 5.8 |
| USC  (Los Angeles, USA) | Los Angeles Cancer Surveillance Program | 274 | 58.9 | ± 11.3 | Population based: Neighbourhood recruits | 587 | 55.9 | ± 12.8 |
| **Total** |  | **3,734** | **58.7** | **± 10.6** |  | **10,067** | **56.5** | **± 11.5** |

a: Cases and controls are non-Hispanic Whites used in discovery and replication analyses only, with the exception of AUS which included a subset of non-Whites used in discovery analysis; cases are serous invasive only (exploratory analysis conducted on additional ethnicities and histologies not included)

b: Age at diagnosis (cases) and age at interview (controls) except for the NHS, which was age at blood draw for both cases and controls; mean and standard deviation based on total number; * indicates no significant difference (*p≥*0.05) between mean age of cases and controls
