# Supplementary material for: Evaluation of Candidate Stromal Epithelial Cross-Talk Genes Identifies Association between Risk of Serous Ovarian Cancer and TERT, a Cancer Susceptibility “Hot-Spot”
Source: PLoS Genet. 2010 Jul 8;6(7):e1001016. doi: 10.1371/journal.pgen.1001016 (PMC2900295; doi:10.1371/journal.pgen.1001016)
Supplement: Table S3 — SNPs successfully genotyped (Illumina & Sequenom) in the discovery stage with PTrend≤0.05 for serous ovarian cancer risk. (0.12 MB DOC) [file pgen.1001016.s004.doc]

Table S3: SNPs successfully genotyped (Illumina & Sequenom) in the discovery stage with *P*_Trend_  ≤ 0.05 for serous ovarian cancer risk

| **Gene symbol** | **CHR #** | **SNPid** | **Minor Allele** | **Major Allele** | **^a^MAF** | **^a^*P*_HWE_** | **^b^OR** | **^b^( 0.95** | **CI)** | **^b^*P*_allelic** | **^c^*P*_trend** |
| --- | --- | --- | --- | --- | --- | --- | --- | --- | --- | --- | --- |
| **PODXL* | 7 | rs1013368 | G | A | 0.34 | 1.00 | 1.32 | (1.14 | - 1.51) | 0.0001126 | 0.0001037 |
| **ITGA6* | 2 | rs13027811 | G | A | 0.12 | 0.87 | 0.68 | (0.54 | - 0.85) | 0.0008275 | 0.0008566 |
| **MMP3* | 11 | rs522616 | G | A | 0.23 | 0.93 | 0.76 | (0.64 | - 0.90) | 0.001178 | 0.001184 |
| **PTTG1* | 5 | rs17057781 | G | A | 0.13 | 0.22 | 1.34 | (1.11 | - 1.62) | 0.002653 | 0.003465 |
| **CSF1* | 1 | rs1999713 | G | A | 0.34 | 0.19 | 1.23 | (1.07 | - 1.41) | 0.003587 | 0.003919 |
| *PLOD2* | 3 | rs1707469 | C | A | 0.34 | 0.32 | 1.22 | (1.06 | - 1.40) | 0.00559 | 0.006242 |
| *SOX9* | 17 | rs6501522 | A | G | 0.02 | 0.73 | 1.74 | (1.16 | - 2.60) | 0.006294 | 0.006677 |
| **TERT* | 5 | rs7726159 | A | C | 0.32 | 0.62 | 1.22 | (1.06 | - 1.40) | 0.006433 | 0.00675 |
| **TIMP3* | 22 | rs5754289 | A | G | 0.17 | 0.54 | 1.26 | (1.06 | - 1.49) | 0.007263 | 0.007529 |
| **MMP26* | 11 | rs11035042 | T | A | 0.11 | 0.00 | 0.73 | (0.58 | - 0.92) | 0.007429 | 0.0076 |
| **FGF2* | 4 | rs17473132 | A | G | 0.06 | 1.00 | 1.41 | (1.09 | - 1.81) | 0.008271 | 0.007884 |
| **ITGA6* | 2 | rs1574028 | A | C | 0.08 | 0.85 | 1.29 | (1.07 | - 1.55) | 0.007741 | 0.008074 |
| *FLT3LG* | 19 | rs3826717 | G | A | 0.08 | 0.40 | 1.35 | (1.08 | - 1.69) | 0.009292 | 0.009067 |
| *OSMR* | 5 | rs10040172 | G | A | 0.19 | 0.55 | 0.78 | (0.65 | - 0.94) | 0.008309 | 0.009422 |
| *MMP3* | 11 | rs650108 | A | G | 0.28 | 0.72 | 0.82 | (0.70 | - 0.95) | 0.01045 | 0.01078 |
| **DDR2* | 1 | rs6693632 | G | A | 0.03 | 0.75 | 1.57 | (1.11 | - 2.22) | 0.01119 | 0.01097 |
| *SPARC* | 5 | rs3756631 | T | A | 0.13 | 0.65 | 1.28 | (1.06 | - 1.56) | 0.01084 | 0.01146 |
| **FN1* | 2 | rs1250229 | A | G | 0.28 | 0.71 | 0.83 | (0.71 | - 0.96) | 0.01475 | 0.01259 |
| **PLOD2* | 3 | rs1512900 | C | G | 0.49 | 1.00 | 0.84 | (0.74 | - 0.96) | 0.0132 | 0.01294 |
| **DDR2* | 1 | rs10917589 | A | G | 0.09 | 0.11 | 0.74 | (0.57 | - 0.95) | 0.01631 | 0.01301 |
| *TGFB2* | 1 | rs10495098 | A | C | 0.38 | 0.58 | 1.19 | (1.04 | - 1.36) | 0.01337 | 0.01395 |
| *ADAM8* | 10 | rs1573041 | A | G | 0.21 | 0.51 | 1.23 | (1.04 | - 1.44) | 0.01372 | 0.01465 |
| *H1F0* | 22 | rs763137 | A | G | 0.12 | 0.90 | 1.28 | (1.05 | - 1.55) | 0.01626 | 0.0149 |
| *PTTG1* | 5 | rs7700446 | A | G | 0.17 | 0.92 | 0.79 | (0.65 | - 0.95) | 0.0132 | 0.01533 |
| *OSMR* | 5 | rs2278324 | A | C | 0.20 | 0.94 | 0.80 | (0.67 | - 0.96) | 0.01515 | 0.01647 |
| **MMP7* | 11 | rs17098236 | A | G | 0.09 | 0.25 | 0.74 | (0.57 | - 0.95) | 0.01864 | 0.01673 |
| *IFI16* | 1 | rs1057024 | G | A | 0.13 | 0.24 | 1.26 | (1.04 | - 1.52) | 0.01687 | 0.01753 |
| *TIMP3* | 22 | rs130290 | A | G | 0.09 | 0.75 | 0.74 | (0.57 | - 0.95) | 0.0184 | 0.01845 |
| *IGFBP4* | 17 | rs2245333 | G | A | 0.32 | 0.33 | 0.84 | (0.72 | - 0.97) | 0.01865 | 0.0186 |
| **PODXL* | 7 | rs4731799 | T | C | 0.49 | 0.54 | 0.88 | (0.79 | - 0.98) | 0.02063 | 0.02015 |
| *FGF2* | 4 | rs167428 | G | A | 0.25 | 1.00 | 1.20 | (1.03 | - 1.39) | 0.02023 | 0.02027 |
| *ITGAV* | 2 | rs11902171 | G | C | 0.27 | 0.28 | 1.19 | (1.03 | - 1.38) | 0.02169 | 0.02057 |
| *IL1R1* | 2 | rs3917332 | T | A | 0.21 | 0.24 | 0.82 | (0.69 | - 0.97) | 0.02175 | 0.02216 |
| *TERT* | 5 | rs11133719 | A | G | 0.17 | 0.88 | 0.81 | (0.67 | - 0.98) | 0.02645 | 0.02479 |
| *VDR* | 12 | rs11574139 | A | T | 0.04 | 1.00 | 0.65 | (0.44 | - 0.96) | 0.0284 | 0.02676 |
| *CD44* | 11 | rs1425802 | G | A | 0.22 | 0.63 | 1.19 | (1.02 | - 1.39) | 0.03048 | 0.02788 |
| **PANX1* | 11 | rs1540177 | A | G | 0.42 | 0.56 | 0.86 | (0.75 | - 0.98) | 0.02926 | 0.02973 |
| **PTEN* | 10 | rs34370136 | A | G | 0.05 | 0.96 | 1.36 | (1.03 | - 1.80) | 0.0307 | 0.0314 |
| *OSMR* | 5 | rs357287 | C | A | 0.31 | 0.92 | 0.85 | (0.73 | - 0.99) | 0.03182 | 0.03172 |
| *MMP1* | 11 | rs7945189 | A | G | 0.09 | 0.96 | 1.28 | (1.03 | - 1.59) | 0.0284 | 0.0319 |
| *CD44* | 11 | rs10836342 | C | G | 0.33 | 0.45 | 0.85 | (0.74 | - 0.99) | 0.03238 | 0.03237 |
| *ITGAV* | 2 | rs3768787 | G | A | 0.22 | 0.17 | 1.19 | (1.01 | - 1.39) | 0.03293 | 0.03342 |
| *MMP14* | 14 | rs12050397 | T | A | 0.17 | 0.26 | 0.82 | (0.68 | - 0.98) | 0.03383 | 0.03425 |
| *MMP1* | 11 | rs514921 | G | A | 0.30 | 0.81 | 0.85 | (0.73 | - 0.99) | 0.03536 | 0.03431 |
| *PODXL* | 7 | rs3735035 | A | G | 0.50 | 0.41 | 0.87 | (0.76 | - 0.99) | 0.03514 | 0.03441 |
| *MMP7* | 11 | rs7935378 | G | A | 0.17 | 0.07 | 0.82 | (0.68 | - 0.99) | 0.03997 | 0.03543 |
| *CTSK* | 1 | rs4379678 | G | A | 0.07 | 0.69 | 1.29 | (1.01 | - 1.65) | 0.03942 | 0.03758 |
| *THBS4* | 5 | rs17879514 | A | G | 0.07 | 0.54 | 0.73 | (0.55 | - 0.98) | 0.03852 | 0.03791 |
| *PODXL* | 7 | rs1477250 | G | A | 0.51 | 0.96 | 0.87 | (0.76 | - 0.99) | 0.03938 | 0.0385 |
| *SAT* | 23 | rs873637 | A | G | 0.07 | 0.24 | 1.30 | (1.01 | - 1.67) | 0.0393 | 0.03851 |
| *VEGF* | 6 | rs3025040 | A | G | 0.13 | 0.09 | 1.22 | (1.01 | - 1.47) | 0.04292 | 0.04018 |
| *CD44* | 11 | rs2295756 | G | A | 0.38 | 0.24 | 0.86 | (0.75 | - 0.99) | 0.0411 | 0.04051 |
| **LCN2* | 9 | rs3814526 | G | A | 0.06 | 0.00 | 0.78 | (0.63 | - 0.98) | 0.03493 | 0.04094 |
| *IGFBP5* | 2 | rs11575194 | A | G | 0.04 | 0.53 | 1.39 | (1.01 | - 1.91) | 0.03972 | 0.04155 |
| **DDR2* | 1 | rs6702820 | G | A | 0.23 | 0.32 | 0.84 | (0.72 | - 0.99) | 0.04228 | 0.04209 |
| **PODXL* | 7 | rs11768640 | A | G | 0.22 | 0.28 | 1.17 | (1.00 | - 1.38) | 0.04592 | 0.04391 |
| *WNT5A* | 3 | rs590386 | A | G | 0.09 | 0.49 | 1.26 | (1.01 | - 1.56) | 0.04168 | 0.04394 |
| *CCL13* | 17 | rs3136675 | A | G | 0.02 | 0.28 | 1.49 | (1.01 | - 2.20) | 0.0417 | 0.04464 |
| *EIF4EBP2* | 10 | rs10999326 | C | G | 0.27 | 0.64 | 0.85 | (0.73 | - 1.00) | 0.0468 | 0.04977 |

a: MAFs and *P*_HWE_ for each SNP determined for controls. SNPs with significant deviations from HWE (0.001<*P*<0.05) included if clustering was optimal

b: Odds ratios, 95% CI and p-values are derived from the allelic test for association using χ^2^ test on 1 df

c: Cochran-Armitage trend test (1df)

* SNPs selected for replication study
