# Supplementary material for: Evaluation of Candidate Stromal Epithelial Cross-Talk Genes Identifies Association between Risk of Serous Ovarian Cancer and TERT, a Cancer Susceptibility “Hot-Spot”
Source: PLoS Genet. 2010 Jul 8;6(7):e1001016. doi: 10.1371/journal.pgen.1001016 (PMC2900295; doi:10.1371/journal.pgen.1001016)
Supplement: Table S4 — Study heterogeneity p-values for serous ovarian cancer risk estimates among non-Hispanic whites for SNPs reported in Table 2. (0.04 MB DOC) [file pgen.1001016.s005.doc]

Table S4: Study heterogeneity *p*-values for serous ovarian cancer risk estimates among non-Hispanic whites for SNPs reported in Table 2

| **Gene** | **SNP** | ***P*_Heterogeneity_** | **Participating OCAC Studies** |
| --- | --- | --- | --- |
| *PODXL* | rs1013368 | 0.79 | AUS, DOV, GER, HAW, HOP, MAL, POL, SEA, STA, UCI, USC, UKO |
| *ITGA6* | rs13027811 | 0.28 | AUS, DOV, HAW, HOP, MAL, MAY, NCO, NEC, NHS, POL, SEA, UCI, UKO, USC |
| *MMP3* | rs522616 | 0.18 | AUS, DOV, GER, HAW, HOP, MAL, MAY, NCO, NEC, NHS, POL, SEA, STA, UCI, UKO, USC |
| *PODXL* | rs11768640 | 0.79 | AUS, MAL, SEA, UKO, USC |
| *PODXL* | rs4731799 | 0.85 | AUS, MAL, SEA, UKO, USC |
| *ITGA6* | rs1574028 | 0.79 | AUS, MAL, SEA, UKO, USC |
| *MMP7* | rs17098236 | 0.12 | AUS, MAL, SEA, UKO, USC |
| *MMP26* | rs11035042 | 0.77 | AUS, MAL, SEA, UKO, USC |
| *FN1* | rs1250229 | 0.39 | AUS, MAL, SEA, UKO, USC |
| *PLOD2* | rs1512900 | 0.70 | AUS, MAL, SEA, UKO, USC |
| *PANX1* | rs1540177 | 0.69 | AUS, MAL, SEA, UKO, USC |
| *PTTG1* | rs17057781 | 0.70 | AUS, MAL, SEA, UKO, USC |
| *CSF1* | rs1999713 | 0.84 | AUS, MAL, SEA, UKO, USC |
| *PTEN* | rs34370136 | 0.95 | AUS, MAL, SEA, UKO, USC |
| *LCN2* | rs3814526 | 0.17 | AUS, MAL, SEA, UKO, USC |
| *TIMP3* | rs5754289 | 0.49 | AUS, MAL, SEA, UKO, USC |
| *DDR2* | rs6693632 | 0.53 | AUS, MAL, SEA, UKO, USC |
| *DDR2* | rs6702820 | 0.91 | AUS, MAL, SEA, UKO, USC |
| *DDR2* | rs10917589 | 0.43 | AUS, MAL, SEA, UKO, USC |
| ***TERT*** | **rs7726159** | **0.005** | **AUS, MAL, SEA, UKO, USC** |
