# Supplementary material for: Evaluation of Candidate Stromal Epithelial Cross-Talk Genes Identifies Association between Risk of Serous Ovarian Cancer and TERT, a Cancer Susceptibility “Hot-Spot”
Source: PLoS Genet. 2010 Jul 8;6(7):e1001016. doi: 10.1371/journal.pgen.1001016 (PMC2900295; doi:10.1371/journal.pgen.1001016)
Supplement: Text S1 — Candidate gene selection and justification. (0.06 MB DOC) [file pgen.1001016.s006.doc]

**Text S1: Candidate gene selection and justification:**

The pathology of cancer tissue invariably includes changes in neighboring cells, generally termed stroma. There is a strong association between morphological features of wounded tissue and inflammation and cancer (elevated levels of infiltrating inflammatory and immune cells) [1,2]. Chronic inflammation is also the most robust epidemiological risk factor of cancer [2-5]. In addition, by examining more than 900 autopsy records of patients with different primary tumors, Paget documented a non-random pattern of metastasis to visceral organs and was struck by the discrepancy between the relative blood supply and the frequency of metastases in certain organs, such as breast and prostate cancer spread to bones. This lead him to suggest what is now termed the “seed and soil” hypothesis, which suggests that local primary stroma supports the cancer cells, and that organ specific metastatic destiny preference is limited by such stromal support[6,7]. Indeed, skin irritation by carcinogen increased the efficiency of carcinogen-unexposed epithelial cell transformation [8]. Even in the context of cells expressing an activated oncogene, tumor formation still depended on wounding [9,10]. This suggested that contrary to the simple cell autonomous DNA damage view suggested by Ames and colleagues [11], carcinogenesis is reliant on higher order cell-cell interactions. We reported expression profiling analysis of 285 ovarian carcinoma, which identified a group of high grade invasive cancer patients with inherently poor prognosis, characterized by a reactive stromal gene expression signature, and extensive desmoplasia [12]. In some instances, the carcinoma-promoting phenotype of fibroblasts residing in breast and prostate cancer microenvironment (carcinoma-associated fibroblasts, CAFs) was demonstrated in xenograft models, which pertain to the inherent and stable basis of this phenotype. Specifically, CAFs lack the ability of normal fibroblasts to attenuate the growth of neighboring transformed epithelial cells [13], and instead can accelerate cancer progression [14-16]. Several hypotheses have been presented for the origin of these altered cells, including standard connective tissue acute phase and stress response [8,17,18], and fibroblast senescence,[19-22] reciprocal interactions with the cancer cells,[23-29] fibroblast specific somatic mutations,[30-34], somatic genomic aberrations [30,32,35], changes of the extracellular matrix density [36], differentiation precursors and infiltrating mesenchymal stem cell,[37,38] (often implicated in metastasis support[38,39]) and inherent genomic predisposition[40,41]. We recently reported that we found no evidence of clonally selected somatic genomic alterations in CAFs[42]. Spontaneous cross talk between epithelial and stromal cells induces the expression of genes in both the stroma and cancer cells [43-51]. These genes include classical cancer stroma markers that reportedly may promote oncogenic potential of adjacent epithelia [12,52-55].

A prominent etiological model for ovarian cancer involves recurrent inflammatory insult to the ovarian surface epithelium [56]. Since cancer is a diseases that largely presents in women at post reproductive age, it is not expected to be selected against in human evolution [57]. By contrast, inflammation, which is an acute response to infection provides evolutionary advantage at reproductive age. Since recovery of the epithelial cell function is critical to wound healing, inflammation activates proto-oncogenes[58], increases genomic instability, via oxidative radicals,[5,59] and protects oncogene-transformed epithelial cells from apoptosis [3,4,60]. In this respect, the stromal response is hardwired in the genome as part of the cancer’s resemblance to a chronic wound, and it is possible that common alleles in human code for improved wound healing, which in later stages in life may also be associated with increased cancer predisposition, due to elevated support from the stroma in premalignant stages. In addition to parsimony, this hypothesis offers clear predictions to scientifically test against corresponding null hypotheses; (1) That co-culture of cancer cells with normal fibroblasts will induce expression of CAF-specific genes in the fibroblasts, [43-51,61] (2) that wounded fibroblasts should promote cancer in a way that is indistinguishable from CAFs [62]. Gene expression profiling of tumor-stromal interactions between co-cultured cancer cells and stromal fibroblasts have previously been performed for cancer cells with the corresponding organ-specific fibroblasts, essentially allowing us to measure which genes operate during wound healing [43-51]. Since we failed to transform normal fibroblasts into CAFs via co-cultivation with cancer cells *in vivo* for extended period of time (Qiu *et al*, in preparation), we turned to the germline variations in human populations, to explore whether alleles in genes that drive or respond to the tumor-stroma interactions in the setting of wound healing, may exhibit increased allelic frequencies in ovarian cancer patients. Many of the genes shown to be activated in these co-cultures are known markers of CAFs *in vivo*, such as *MMP1*, *MMP3*, collagens, *TNC*, etc. We therefore compiled a candidate gene list that is composed of either genes that are upregulated in epithelial-fibroblast co-cultures or expressed in ovarian cancer [12,63-67]. In addition, we included genes that are likely drivers of the co-culture response *in vivo*, such as proteases that degrade the basement membrane barrier, intercepting between those two cell types in normal tissue, such as gap junction proteins [68,69] tPA, uPA and ADAM8. The candidate drivers were further selected, based on genes expression showing strong correlation with the overall co-culture gene expression across 765 carcinoma profiles (Haviv *et al*, in preparation). Since our hypothesis was that the cross talk modifies critical aspects of epithelial transformation, some critical surrogate markers of such epithelial processes, such as proliferation markers (BuB1 and CCND2)[70], angiogenesis,[15,16] epithelial stem cell markers (TERT[71], CD24 and CD44[72]), metastatic potential [39,73] or cross talk with other components of the stroma (MMP9, CSF1 and AREG) [74-78] were also included. Specific justification of each of the interrogated candidate genes is included in Table S1.

In addition to those derived from co-culture experiments, we explored candidate genes that are regulated during the estrous cycle in ovarian surface epithelial cells (OSE) *in vivo*. We have previously reported global gene expression changes in pure populations of normal mouse OSE from immature mice (low hormone levels), cycling mice at proestrus evening (high hormone levels just prior to ovulation), and at estrus morning (low hormone levels just after ovulation) [67]. The majority of transcripts (n=161) were up-regulated on proestrus evening, just prior to ovulation, co-incident with the surge in ovulatory hormones. Ovulatory hormones and a high number of life-time ovulations, with concomitant cell proliferation, apoptosis, wound-repair and inflammation, have been associated with ovarian cancer increased risk. We therefore analysed the estrous regulated gene set to identify genes that are mutated in human cancer or differentially expressed between normal human ovarian cells and ovarian cancer, in our own and other publically available datasets [12,65,79-83]. We identified over 300 genes that are regulated in the normal mouse OSE during the estrous cycle and consistently dysregulated or mutated in human ovarian carcinoma (Emmanuel *et al*, manuscript in preparation). Candidates for SNP analysis were selected based on consistently between expression array datasets and literature evidence for a potential role in oncogenic processes.

**References**

1. Virchow RL (1966) Rudolph Virchow on ochronosis.1866. Arthritis Rheum 9: 66-71.

2. Balkwill F, Mantovani A (2001) Inflammation and cancer: back to Virchow? Lancet 357: 539-545.

3. Karin M, Lawrence T, Nizet V (2006) Innate immunity gone awry: linking microbial infections to chronic inflammation and cancer. Cell 124: 823-835.

4. Lin WW, Karin M (2007) A cytokine-mediated link between innate immunity, inflammation, and cancer. J Clin Invest 117: 1175-1183.

5. Mantovani A, Allavena P, Sica A, Balkwill F (2008) Cancer-related inflammation. Nature 454: 436-444.

6. Paget S (1889) The distributions of secondary growths in cancer of the breast. Lancet 1: 571-573.

7. Fidler IJ, Yano S, Zhang RD, Fujimaki T, Bucana CD (2002) The seed and soil hypothesis: vascularisation and brain metastases. Lancet Oncol 3: 53-57.

8. Billingham RE, Orr JW, Woodhouse DL (1951) Transplantation of skin components during chemical carcinogenesis with 20-methylcholanthrene. Br J Cancer 5: 417-432.

9. Dolberg DS, Hollingsworth R, Hertle M, Bissell MJ (1985) Wounding and its role in RSV-mediated tumor formation. Science 230: 676-678.

10. Dolberg DS, Bissell MJ (1984) Inability of Rous sarcoma virus to cause sarcomas in the avian embryo. Nature 309: 552-556.

11. Ames BN, Gurney EG, Miller JA, Bartsch H (1972) Carcinogens as frameshift mutagens: metabolites and derivatives of 2-acetylaminofluorene and other aromatic amine carcinogens. Proc Natl Acad Sci U S A 69: 3128-3132.

12. Tothill RW, Tinker AV, George J, Brown R, Fox SB, et al. (2008) Novel molecular subtypes of serous and endometrioid ovarian cancer linked to clinical outcome. Clin Cancer Res 14: 5198-5208.

13. Sadlonova A, Novak Z, Johnson MR, Bowe DB, Gault SR, et al. (2005) Breast fibroblasts modulate epithelial cell proliferation in three-dimensional in vitro co-culture. Breast Cancer Res 7: R46-59.

14. Orimo A, Gupta PB, Sgroi DC, Arenzana-Seisdedos F, Delaunay T, et al. (2005) Stromal Fibroblasts Present in Invasive Human Breast Carcinomas Promote Tumor Growth and Angiogenesis through Elevated SDF-1/CXCL12 Secretion. Cell 121: 335-348

15. Olumi AF, Grossfeld GD, Hayward SW, Carroll PR, Tlsty TD, et al. (1999) Carcinoma-associated fibroblasts direct tumor progression of initiated human prostatic epithelium. Cancer Res 59: 5002-5011.

16. Orimo A, Weinberg RA (2006) Stromal fibroblasts in cancer: a novel tumor-promoting cell type. Cell Cycle 5: 1597-1601.

17. Kuperwasser C, Chavarria T, Wu M, Magrane G, Gray JW, et al. (2004) Reconstruction of functionally normal and malignant human breast tissues in mice. Proc Natl Acad Sci U S A 101: 4966-4971.

18. Maffini MV, Soto AM, Calabro JM, Ucci AA, Sonnenschein C (2004) The stroma as a crucial target in rat mammary gland carcinogenesis. J Cell Sci 117: 1495-1502.

19. Campisi J (2004) Fragile fugue: p53 in aging, cancer and IGF signaling. Nat Med 10: 231-232.

20. Campisi J (2005) Aging, tumor suppression and cancer: high wire-act! Mech Ageing Dev 126: 51-58.

21. Krtolica A, Campisi J (2002) Cancer and aging: a model for the cancer promoting effects of the aging stroma. Int J Biochem Cell Biol 34: 1401-1414.

22. Parrinello S, Coppe JP, Krtolica A, Campisi J (2005) Stromal-epithelial interactions in aging and cancer: senescent fibroblasts alter epithelial cell differentiation. J Cell Sci 118: 485-496.

23. Bierie B, Moses HL (2006) Tumour microenvironment: TGFbeta: the molecular Jekyll and Hyde of cancer. Nat Rev Cancer 6: 506-520.

24. Ellis MJ, Singer C, Hornby A, Rasmussen A, Cullen KJ (1994) Insulin-like growth factor mediated stromal-epithelial interactions in human breast cancer. Breast Cancer Res Treat 31: 249-261.

25. Matsumoto K, Nakamura T (2006) Hepatocyte growth factor and the Met system as a mediator of tumor-stromal interactions. Int J Cancer 119: 477-483.

26. Ostman A (2004) PDGF receptors-mediators of autocrine tumor growth and regulators of tumor vasculature and stroma. Cytokine Growth Factor Rev 15: 275-286.

27. Wisniewski HG, Hua JC, Poppers DM, Naime D, Vilcek J, et al. (1996) TNF/IL-1-inducible protein TSG-6 potentiates plasmin inhibition by inter-alpha-inhibitor and exerts a strong anti-inflammatory effect in vivo. J Immunol 156: 1609-1615.

28. Joesting MS, Perrin S, Elenbaas B, Fawell SE, Rubin JS, et al. (2005) Identification of SFRP1 as a candidate mediator of stromal-to-epithelial signaling in prostate cancer. Cancer Res 65: 10423-10430.

29. Bernards R, Weinberg RA (2002) A progression puzzle. Nature 418: 823.

30. Moinfar F, Man YG, Arnould L, Bratthauer GL, Ratschek M, et al. (2000) Concurrent and independent genetic alterations in the stromal and epithelial cells of mammary carcinoma: implications for tumorigenesis. Cancer Res 60: 2562-2566.

31. Fukino K, Shen L, Matsumoto S, Morrison CD, Mutter GL, et al. (2004) Combined total genome loss of heterozygosity scan of breast cancer stroma and epithelium reveals multiplicity of stromal targets. Cancer Res 64: 7231-7236.

32. Kurose K, Gilley K, Matsumoto S, Watson PH, Zhou XP, et al. (2002) Frequent somatic mutations in PTEN and TP53 are mutually exclusive in the stroma of breast carcinomas. Nat Genet 32: 355-357.

33. Plon SE, Pirics ML, Nuchtern J, Hicks J, Russell H, et al. (2008) Multiple tumors in a child with germ-line mutations in TP53 and PTEN. N Engl J Med 359: 537-539.

34. Patocs A, Zhang L, Xu Y, Weber F, Caldes T, et al. (2007) Breast-cancer stromal cells with TP53 mutations and nodal metastases. N Engl J Med 357: 2543-2551.

35. Tuhkanen H, Anttila M, Kosma VM, Yla-Herttuala S, Heinonen S, et al. (2004) Genetic alterations in the peritumoral stromal cells of malignant and borderline epithelial ovarian tumors as indicated by allelic imbalance on chromosome 3p. Int J Cancer 109: 247-252.

36. Paszek MJ, Zahir N, Johnson KR, Lakins JN, Rozenberg GI, et al. (2005) Tensional homeostasis and the malignant phenotype. Cancer Cell 8: 241-254.

37. Studeny M, Marini FC, Dembinski JL, Zompetta C, Cabreira-Hansen M, et al. (2004) Mesenchymal stem cells: potential precursors for tumor stroma and targeted-delivery vehicles for anticancer agents. J Natl Cancer Inst 96: 1593-1603.

38. Karnoub AE, Dash AB, Vo AP, Sullivan A, Brooks MW, et al. (2007) Mesenchymal stem cells within tumour stroma promote breast cancer metastasis. Nature 449: 557-563.

39. Eckhardt BL, Parker BS, van Laar RK, Restall CM, Natoli AL, et al. (2005) Genomic analysis of a spontaneous model of breast cancer metastasis to bone reveals a role for the extracellular matrix. Mol Cancer Res 3: 1-13.

40. Deshpande A, Nolan JP, White PS, Valdez YE, Hunt WC, et al. (2005) TNF-alpha promoter polymorphisms and susceptibility to human papillomavirus 16-associated cervical cancer. J Infect Dis 191: 969-976.

41. Morgan GJ, Adamson PJ, Mensah FK, Spink CF, Law GR, et al. (2005) Haplotypes in the tumour necrosis factor region and myeloma. Br J Haematol 129: 358-365.

42. Qiu W, Sridhar A, Opeskin K, Fox S, Hu M, et al. (2008) No evidence of genomic alterations in cancer associated fibroblasts from human breast and ovarian carcinomas. Nat Genet In Press.

43. Buess M, Nuyten DS, Hastie T, Nielsen T, Pesich R, et al. (2007) Characterization of heterotypic interaction effects in vitro to deconvolute global gene expression profiles in cancer. Genome Biol 8: R191.

44. Fromigue O, Louis K, Dayem M, Milanini J, Pages G, et al. (2003) Gene expression profiling of normal human pulmonary fibroblasts following coculture with non-small-cell lung cancer cells reveals alterations related to matrix degradation, angiogenesis, cell growth and survival. Oncogene 22: 8487-8497.

45. Gallagher PG, Bao Y, Prorock A, Zigrino P, Nischt R, et al. (2005) Gene expression profiling reveals cross-talk between melanoma and fibroblasts: implications for host-tumor interactions in metastasis. Cancer Res 65: 4134-4146.

46. Sato N, Maehara N, Goggins M (2004) Gene expression profiling of tumor-stromal interactions between pancreatic cancer cells and stromal fibroblasts. Cancer Res 64: 6950-6956.

47. Lacina L, Dvorankova B, Smetana K, Jr., Chovanec M, Plzak J, et al. (2007) Marker profiling of normal keratinocytes identifies the stroma from squamous cell carcinoma of the oral cavity as a modulatory microenvironment in co-culture. Int J Radiat Biol 83: 837-848.

48. Miki Y, Suzuki T, Tazawa C, Yamaguchi Y, Kitada K, et al. (2007) Aromatase localization in human breast cancer tissues: possible interactions between intratumoral stromal and parenchymal cells. Cancer Res 67: 3945-3954.

49. Mahadevan D, Von Hoff DD (2007) Tumor-stroma interactions in pancreatic ductal adenocarcinoma. Mol Cancer Ther 6: 1186-1197.

50. Wang J, Levenson AS, Satcher RL, Jr. (2006) Identification of a unique set of genes altered during cell-cell contact in an in vitro model of prostate cancer bone metastasis. Int J Mol Med 17: 849-856.

51. Bavik C, Coleman I, Dean JP, Knudsen B, Plymate S, et al. (2006) The gene expression program of prostate fibroblast senescence modulates neoplastic epithelial cell proliferation through paracrine mechanisms. Cancer Res 66: 794-802.

52. Rich JN, Hans C, Jones B, Iversen ES, McLendon RE, et al. (2005) Gene expression profiling and genetic markers in glioblastoma survival. Cancer Res 65: 4051-4058.

53. Spentzos D, Levine DA, Ramoni MF, Joseph M, Gu X, et al. (2004) A Gene Expression Signature With Independent Prognostic Significance in Epithelial Ovarian Cancer. J Clin Oncol: JCO.2004.2004.2070.

54. Inaguma Y, Kusakabe M, Mackie EJ, Pearson CA, Chiquet-Ehrismann R, et al. (1988) Epithelial induction of stromal tenascin in the mouse mammary gland: from embryogenesis to carcinogenesis. Dev Biol 128: 245-255.

55. Paley PJ, Goff BA, Gown AM, Greer BE, Sage EH (2000) Alterations in SPARC and VEGF immunoreactivity in epithelial ovarian cancer. Gynecol Oncol 78: 336-341.

56. Fleming JS, Beaugie CR, Haviv I, Chenevix-Trench G, Tan OL (2006) Incessant ovulation, inflammation and epithelial ovarian carcinogenesis: revisiting old hypotheses. Mol Cell Endocrinol 247: 4-21.

57. Sommer SS (1994) Does cancer kill the individual and save the species? Hum Mutat 3: 166-169.

58. Pollard JW (2008) Macrophages define the invasive microenvironment in breast cancer. J Leukoc Biol 84: 623-630.

59. Radisky DC, Levy DD, Littlepage LE, Liu H, Nelson CM, et al. (2005) Rac1b and reactive oxygen species mediate MMP-3-induced EMT and genomic instability. Nature 436: 123-127.

60. Pikarsky E, Porat RM, Stein I, Abramovitch R, Amit S, et al. (2004) NF-[kappa]B functions as a tumour promoter in inflammation-associated cancer. Nature 431: 461-466.

61. Shipitsin M, Campbell LL, Argani P, Weremowicz S, Bloushtain-Qimron N, et al. (2007) Molecular definition of breast tumor heterogeneity. Cancer Cell 11: 259-273.

62. Hu M, Yao J, Carroll DK, Weremowicz S, Chen H, et al. (2008) Regulation of in situ to invasive breast carcinoma transition. Cancer Cell 13: 394-406.

63. Jazaeri AA, Yee CJ, Sotiriou C, Brantley KR, Boyd J, et al. (2002) Gene Expression Profiles of BRCA1-Linked, BRCA2-Linked, and Sporadic Ovarian Cancers. J Natl Cancer Inst 94: 990-1000.

64. Spentzos D, Levine DA, Kolia S, Otu H, Boyd J, et al. (2005) Unique gene expression profile based on pathologic response in epithelial ovarian cancer. J Clin Oncol 23: 7911-7918.

65. Donninger H, Bonome T, Radonovich M, Pise-Masison CA, Brady J, et al. (2004) Whole genome expression profiling of advance stage papillary serous ovarian cancer reveals activated pathways. Oncogene 23: 8065-8077.

66. Boussioutas A, Li H, Liu J, Waring P, Lade S, et al. (2003) Distinctive patterns of gene expression in premalignant gastric mucosa and gastric cancer. Cancer Res 63: 2569-2577.

67. Gava N, C LC, Bye C, Byth K, deFazio A (2008) Global gene expression profiles of ovarian surface epithelial cells in vivo. J Mol Endocrinol 40: 281-296.

68. Woodward TL, Sia MA, Blaschuk OW, Turner JD, Laird DW (1998) Deficient epithelial-fibroblast heterocellular gap junction communication can be overcome by co-culture with an intermediate cell type but not by E-cadherin transgene expression. J Cell Sci 111: 3529-3539.

69. Stuhlmann D, Ale-Agha N, Reinehr R, Steinbrenner H, Ramos MC, et al. (2003) Modulation of homologous gap junctional intercellular communication of human dermal fibroblasts via a paracrine factor(s) generated by squamous tumor cells. Carcinogenesis 24: 1737-1748.

70. Shedden K, Cooper S (2002) Analysis of cell-cycle-specific gene expression in human cells as determined by microarrays and double-thymidine block synchronization. Proc Natl Acad Sci U S A 99: 4379-4384.

71. Campisi J (2005) Suppressing cancer: the importance of being senescent. Science 309: 886-887.

72. Allinen M, Beroukhim R, Cai L, Brennan C, Lahti-Domenici J, et al. (2004) Molecular characterization of the tumor microenvironment in breast cancer. Cancer Cell 6: 17-32.

73. Xie D, Nakachi K, Wang H, Elashoff R, Koeffler HP (2001) Elevated Levels of Connective Tissue Growth Factor, WISP-1, and CYR61 in Primary Breast Cancers Associated with More Advanced Features. Cancer Res 61: 8917-8923.

74. Huang S, Van Arsdall M, Tedjarati S, McCarty M, Wu W, et al. (2002) Contributions of stromal metalloproteinase-9 to angiogenesis and growth of human ovarian carcinoma in mice. J Natl Cancer Inst 94: 1134-1142.

75. Zhang L, Conejo-Garcia JR, Katsaros D, Gimotty PA, Massobrio M, et al. (2003) Intratumoral T cells, recurrence, and survival in epithelial ovarian cancer. N Engl J Med 348: 203-213.

76. Sternlicht MD, Sunnarborg SW, Kouros-Mehr H, Yu Y, Lee DC, et al. (2005) Mammary ductal morphogenesis requires paracrine activation of stromal EGFR via ADAM17-dependent shedding of epithelial amphiregulin. Development 132: 3923-3933.

77. Lin EY, Nguyen AV, Russell RG, Pollard JW (2001) Colony-stimulating factor 1 promotes progression of mammary tumors to malignancy. J Exp Med 193: 727-740.

78. Buscher M, Rahmsdorf HJ, Litfin M, Karin M, Herrlich P (1988) Activation of the c-fos gene by UV and phorbol ester: different signal transduction pathways converge to the same enhancer element. Oncogene 3: 301-311.

79. Bonome T, Lee JY, Park DC, Radonovich M, Pise-Masison C, et al. (2005) Expression profiling of serous low malignant potential, low-grade, and high-grade tumors of the ovary. Cancer Res 65: 10602-10612.

80. Futreal PA, Coin L, Marshall M, Down T, Hubbard T, et al. (2004) A census of human cancer genes. Nat Rev Cancer 4: 177-183.

81. Greenman C, Stephens P, Smith R, Dalgliesh GL, Hunter C, et al. (2007) Patterns of somatic mutation in human cancer genomes. Nature 446: 153-158.

82. Heinzelmann-Schwarz VA, Gardiner-Garden M, Henshall SM, Scurry J, Scolyer RA, et al. (2004) Overexpression of the cell adhesion molecules DDR1, Claudin 3, and Ep-CAM in metaplastic ovarian epithelium and ovarian cancer. Clin Cancer Res 10: 4427-4436.

83. Lu KH, Patterson AP, Wang L, Marquez RT, Atkinson EN, et al. (2004) Selection of potential markers for epithelial ovarian cancer with gene expression arrays and recursive descent partition analysis. Clin Cancer Res 10: 3291-3300.

84. Zhong L, Roybal J, Chaerkady R, Zhang W, Choi K, et al. (2008) Identification of secreted proteins that mediate cell-cell interactions in an in vitro model of the lung cancer microenvironment. Cancer Res 68: 7237-7245.

85. Diaw L, Roth M, Schwinn DA, d'Alelio ME, Green LJ, et al. (2005) Characteristics of a human prostate stromal cell line related to its use in a stromal-epithelial coculture model for the study of cancer chemoprevention. In Vitro Cell Dev Biol Anim 41: 142-148.
